# Review on the Mechanism and Performance Enhancement of Biomass-Based Rejuvenators in Reclaimed Asphalt Recycling

**DOI:** 10.3390/polym18050585

**Published:** 2026-02-27

**Authors:** Renqing Wang, Xiule Chen, Peifeng Cheng

**Affiliations:** School of Civil Engineering and Transportation, Northeast Forestry University, Harbin 150040, China; wrq2257569727@outlook.com (R.W.);

**Keywords:** biomass-based rejuvenator, aged asphalt, rejuvenation mechanism, performance, low-carbon environmental protection

## Abstract

Against the backdrop of the continuous advancement of high-quality development in road infrastructure and the growing demand for waste asphalt recycling, the application limitations of traditional petroleum-based asphalt rejuvenators have become increasingly prominent due to their high resource dependence, poor compatibility with aged asphalt, and high volatility. By contrast, bio-oil, characterized by wide feedstock availability, outstanding renewability, and the inherent potential to modulate the colloidal structure and properties of aged asphalt, has gradually emerged as a critical research direction in the field of asphalt rejuvenator development. This paper provides a comprehensive review on the research, development and engineering application of bio-based rejuvenators. Firstly, the main feedstock systems, including vegetable oils, lignin derivatives and algal oils, are introduced, and the core preparation technologies (e.g., pyrolysis and alkali-catalyzed transesterification) are discussed, along with the impacts of their key process parameters on the chemical composition and storage stability of the end products. Subsequently, the performance of various bio-based rejuvenators in optimizing the rheological properties, high- and low-temperature performance, as well as fatigue and cracking resistance of aged asphalt is summarized, and the underlying rejuvenation mechanisms are generalized. Finally, the prevailing technical bottlenecks, such as inconsistent quality of as-prepared products and insufficient understanding of the long-term aging mechanism, are analyzed. Future research directions including oriented molecular modification, interfacial regulation, and full life cycle assessment (LCA) are proposed, to provide a systematic reference for their large-scale engineering application.

## 1. Introduction

Asphalt pavement holds a dominant position in China’s transportation infrastructure, owing to its superior driving comfort, low traffic noise, and convenient maintenance. However, under the coupled effects of traffic loading, cyclic temperature fluctuations, and moisture erosion, asphalt is prone to aging-induced damage including oxidation, hardening, and crack propagation, which further leads to the deterioration of pavement performance and a significant reduction in service life [1,2,3].

To quantify the aging degree of asphalt, Hou and Wang established a multi-dimensional aging evaluation index system covering physical properties, rheological characteristics, and micro-scale composition. Accurate assessment of asphalt aging status was achieved via the correlation model between the established indices and aging degree, which provides a theoretical basis for determining the optimal pavement maintenance timing. The spectrophotometry method introduced in their study quantified the correlation between asphaltene content and absorbance, thus verifying the reliability of the proposed index system. This system, which integrates traditional testing methods and emerging spectroscopic techniques, has promoted the development of asphalt aging evaluation towards a multi-scale and quantitative direction [4,5,6].

Asphalt damage repair can be categorized into two types: intrinsic self-healing of the material and intervention with exogenous rejuvenators. He et al. summarized the key influencing factors (including material properties, environmental conditions, and technical parameters) and mainstream evaluation methods (including macro performance characterization, micro-structure analysis, and energy evolution assessment) of asphalt self-healing behavior, which provides a critical reference for the optimization of asphalt self-healing technologies [7,8]. The study conducted by Bueno demonstrated that induction heating technology, which realizes heating by incorporating conductive-magnetic materials and utilizing an alternating electromagnetic field, can effectively induce micro-crack closure and restore the stiffness of asphalt pavement. Full-scale test results verified that this technology can extend the service life of asphalt pavement by more than 30%, and the service life can be nearly doubled after multiple healing cycles [9].

Asphalt recycling technology has become the core measure for sustainable pavement rehabilitation and recycling. Both laboratory and field studies have confirmed that asphalt rejuvenators can significantly improve the rheological properties and cracking resistance of aged asphalt and present great potential for engineering popularization. Meanwhile, the application of rejuvenators can effectively reduce the consumption of virgin asphalt and the accumulation of waste pavement materials [10,11,12,13,14]. Although petroleum-based rejuvenators are widely used in current engineering practices, their application is severely restricted by inherent drawbacks, including high dependence on non-renewable petroleum resources, high volatility at elevated temperatures, and non-negligible risk of secondary oxidation. In addition, high-temperature treatment of waste engine oil-based rejuvenators may generate hazardous emissions during production and application [15,16], which further limits their sustainable application. With the increasing supply pressure of crude oil and the advancement of China’s “Dual Carbon” strategic goals, the development of clean and controllable asphalt rejuvenation systems has become an industry-wide consensus [17,18,19,20].

Represented by vegetable oils, lignin derivatives, and algal oils, bio-based rejuvenators exhibit prominent advantages such as renewability, low environmental impact, and high flexibility for targeted chemical modification, and can effectively restore the comprehensive properties of aged asphalt [21,22,23,24]. The feedstocks of bio-based rejuvenators are mainly derived from agricultural and forestry wastes and food industry by-products, endowing them with better economic feasibility in large-scale production compared with petroleum-based systems. Advanced technologies such as pyrolysis have greatly improved the preparation efficiency and storage stability of the products [25,26,27], and the application of bio-based rejuvenators also presents additional benefits in terms of pollution control and carbon emission reduction [28].

**Figure 1:**
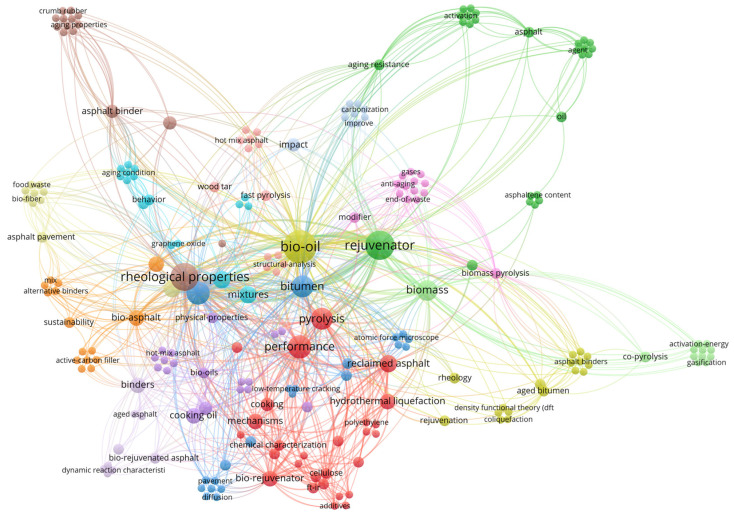
Hotspot Analysis of Bio-based Rejuvenators.

**Figure 2:**
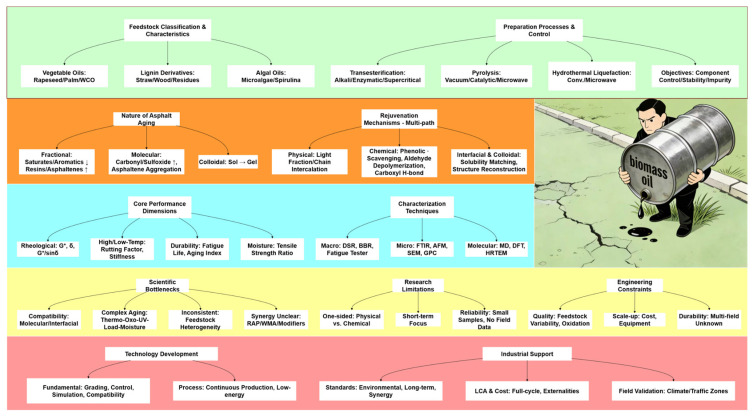
The Framework of the Article.

On this basis, this paper presents a systematic review of the key technologies of bio-based rejuvenators in waste asphalt recycling, with a focus on the following aspects: feedstock sources and preparation processes, chemical composition and physicochemical properties, the necessity of asphalt aging control and rejuvenation, performance improvement effects on aged asphalt, underlying rejuvenation mechanisms, as well as the key scientific issues and challenges restricting their engineering application. Finally, future research directions including oriented molecular regulation and interfacial behavior modeling are proposed, to promote the translation of bio-based rejuvenators from laboratory research to engineering practice and provide technical support for the recycling of road materials and the low-carbon development of road infrastructure. We present Figure 1 herein, which clearly illustrates the overall research network and frontiers centered on bio-oil, covering its pyrolytic preparation, performance regulation of asphalt as a rejuvenator (with a particular focus on rheological properties), and its application in reclaimed asphalt pavement. Meanwhile, the conceptual framework of this study is provided in Figure 2. The figure systematically establishes the full-chain research framework of bio-oil-based asphalt rejuvenation technology, which covers the feedstock classification and preparation processes of bio-oil, the nature of asphalt aging and multi-path rejuvenation mechanisms, the performance evaluation dimensions and multi-scale characterization techniques of rejuvenated asphalt, the existing scientific bottlenecks and engineering constraints in this field, as well as the core directions for future technological development and industrial support to facilitate readers’ comprehensive understanding of the research context.

## 2. Critical Knowledge Gaps in Bio-Oil for Asphalt Rejuvenation

### 2.1. Abnormal High-Temperature Performance of Bio-Oil Modified Asphalt and Its Underlying Mechanism

The diverse feedstock sources and complex chemical composition of bio-oil, the core component of bio-based asphalt rejuvenators, are the key triggers for the contradictory high-temperature performance of modified asphalt. However, the specific underlying action mechanism remains unclear, which constitutes a prominent critical knowledge gap in this field. Even if bio-oils produced from different biomass feedstocks via thermochemical conversion have similar appearance and basic physicochemical properties, there are still significant differences in their content of oxygen-containing organic compounds, proportion of light fractions, and impurity levels. Some bio-oils, rich in polar components such as phenolic and ketonic compounds, can effectively improve the high-temperature stability of aged asphalt. In contrast, bio-oils from other sources with high contents of low-boiling-point components and residual moisture will reduce the high-temperature viscosity of asphalt after incorporation, leading to an increased risk of pavement distresses such as rutting. Meanwhile, discrepancies in the refining processes of bio-oil further exacerbate this contradiction. For bio-oils without deep deacidification and dehydration treatment, the contained organic acid components will damage the molecular structure of asphalt binder, further amplifying the instability of high-temperature performance. However, there is still a lack of in-depth research on the matching rules between bio-oils with varying component characteristics and the high-temperature compatibility of asphalt binders.

### 2.2. Identification of Dominant Functional Groups for Anti-Aging Performance

The short-term softening effect of bio-oil on aged asphalt, namely performance recovery achieved by supplementing light fractions and diluting macromolecules in aged asphalt. However, there is insufficient research on the identification of dominant functional groups for long-term anti-aging performance and their corresponding action mechanisms. The essence of asphalt aging is the oxidative cracking and restructuring of asphalt molecules, accompanied by the generation of aging-related functional groups including carbonyl (C=O) and sulfoxide (S=O) groups. Although functional groups such as alkanes, aromatics, and phenols contained in bio-oil can temporarily supplement the light fractions of asphalt and achieve short-term softening, not all of these functional groups can inhibit the long-term oxidative aging of asphalt. At present, there is no clear distinction between short-term softening functional groups and long-term anti-aging functional groups. For example, whether phenolic functional groups can achieve long-term anti-aging effects by inhibiting free radical oxidation, how the ratio of alkane to aromatic functional groups affects the durability of anti-aging performance, and the stability evolution of different functional groups under long-term thermo-oxidative and ultraviolet (UV) irradiation remain unresolved. The knowledge gaps in these key issues prevent the targeted preparation of long-acting bio-based asphalt rejuvenators [29].

### 2.3. Discrepancy Between Laboratory and Field Performance

Most laboratory studies adopt standardized aging and rejuvenation simulation protocols with strictly controlled conditions including temperature, humidity, and loading, which can only simulate single or a limited number of aging factors, and fail to replicate the complex multi-factor coupling effects in the field environment. These include the long-term synergistic effects of thermo-oxidation, UV irradiation, heavy traffic loading, and moisture erosion, as well as the restriction on the diffusion and action efficiency of rejuvenators imposed by micro-defects such as pavement cracks [30]. Meanwhile, laboratory tests mostly focus on the short-term physicochemical indexes of rejuvenated asphalt, while ignoring the long-term diffusion uniformity of rejuvenators in the field, the interfacial bonding stability with asphalt mixture, and the deterioration reactions such as volatilization and polymerization of bio-oil components during long-term service. In addition, traditional laboratory testing methods have inherent limitations including time-effect lag and insufficient spatial coverage, which cannot accurately capture the real-time aging state of asphalt in the field, further exacerbating the discrepancy between laboratory performance and field durability. However, the quantitative evaluation and correction mechanisms for this discrepancy have not yet been well established.

## 3. Source and Preparation Process of Biomass-Based Rejuvenator

### 3.1. Vegetable Oils

Vegetable oil-based feedstocks have a wide range of sources, including large-scale agriculturally cultivated oil crops such as rapeseed and palm, as well as waste cooking oil (WCO), which offer the inherent advantages of renewability and resource circularity [31]. For rapeseed oil-based rejuvenators, Table 1 systematically summarizes the primary preparation processes and key process parameters of rapeseed oil-derived biodiesel. As detailed in Table 1, the summary covers various mainstream technical routes, including alkaline-catalyzed transesterification, enzymatic transesterification, supercritical methanol transesterification, and the extraction-transesterification coupled process. By comparing the catalyst type and dosage, reaction temperature and duration, methanol-to-oil molar ratio, and final product yield or conversion rate across different studies, it provides a straightforward, multi-dimensional data reference and comparative basis for the process selection and parameter optimization of rapeseed oil-based biodiesel for asphalt rejuvenation applications.

In research focused on palm oil, Thiam et al. used palm oil and palm by-product biomass (including husks, fibers, and other residues) as feedstocks to prepare biodiesel via technical routes such as heterogeneous catalysis and fast pyrolysis and identified the optimal reaction conditions [32]. Wu et al. concentrated on the resource utilization of oil palm waste. They conducted on-site sampling of waste streams including empty fruit bunches (EFB), mesocarp fibers (MF), kernel shells (KS), and palm oil mill effluent (POME), systematically analyzed their key properties including calorific value, elemental composition, and biochemical methane potential (BMP), and optimized production parameters through software simulation. This work ultimately achieved the goal of reducing key environmental indicators such as external sulfur emissions [33].

In addition, Soojin et al. addressed the limitations of traditional alkaline-catalyzed processes imposed by free fatty acids (FFA) and moisture in waste vegetable oil by adopting a supercritical methanol system to realize simultaneous transesterification and esterification reactions. They calibrated the boiling point parameters of triolein through thermogravimetric analysis (TGA) to improve the accuracy of HYSYS simulation. Under the optimized conditions of a reaction temperature of 350 °C, pressure of 19 MPa, methanol-to-oil molar ratio of 24:1, and reaction time of 13.3 min, a triolein conversion rate of 96% was achieved, and the purity of the as-prepared biodiesel reached 99.8 wt%, which fully complies with the EN 14214 standard. This catalyst-free process avoids saponification side reactions and product emulsification issues, and achieves efficient methanol recovery via a flash evaporator, thus exhibiting significantly superior energy efficiency and economic feasibility compared with the traditional alkaline-catalyzed process [34].

**Table 1:** Preparation process parameters of rapeseed oil-based biodiesel.

Researcher	Production Technology	Processing Parameters	Ratio of Methanol to Oil
Rashid et al. [35]	Alkaline-catalyzed transesterification of rapeseed oil	1.0% KOH, 65 °C, 600 rpm; yield 95–96%	6
Yuan et al. [36]	Two-step process for high FFA waste rapeseed oil (pretreatment + alkali-catalyzed transesterification)	Pre-treatment to reduce FFA to <2%; optimal catalyst at 1.0%, 65.4 min, 48.2 °C; conversion rate of 83.34%	6.5
Jang et al. [37]	Two-step enzymatic method for crude rapeseed oil (phospholipase A_2_ degumming + immobilized lipase transesterification)	Degumming: phospholipase A_2_ (50 ppm) at 50 °C for 2 h, phospholipid decreased from 138.8 ppm to 4.35 ppm; optimal conditions: immobilized enzyme 24.4%, buffer 13.5%, methanol 15.8%; conversion rate 88.9%	6
Qiu et al. [38]	Alkali-catalyzed transesterification of soybean oil-canola oil blend	Optimal conditions: NaOH 0.8%, 55 °C, 2 h; yield 94%	5
Saka et al. [39]	Rapeseed oil supercritical methanol transesterification	350–400 °C, 45–65 MPa, 240 s; yield > 95%	42
Shi et al. [40]	Rapeseed oil two-phase solvent extraction (TSE)—transesterification coupling	Optimal conditions: NaOH 1.1%, 120 min, 55–60 °C; conversion rate 98.2%	9
Boz et al. [41]	Heterogeneous catalyzed transesterification of rapeseed oil using nano-γ-Al_2_O_3_ loaded with KF	773 K calcination; optimal conditions: catalyst 3%, 338 K, 8 h; conversion rate 97.7%	15

### 3.2. Lignin and Lignin Derivatives

Lignin, a core structural component of lignocellulosic agricultural and forestry wastes including straw, wood, and bamboo chips, is a naturally occurring renewable organic polymer. It is primarily generated as a by-product of agricultural and forestry product processing and thus presents prominent advantages for resource circularity and waste valorization.

With respect to the production of lignin-derived bio-oil, Fan et al. prepared bio-oil through vacuum pyrolysis of rapeseed straw feedstock and optimized the process conditions via numerical simulation in MATLAB. The results demonstrated that a bio-oil yield ranging from 43.15% to 43.44% was achieved under the optimized conditions: a pyrolysis temperature of approximately 495.5 °C, a heating rate of 19.4 °C/min, a reaction pressure of 5.0 kPa, and a holding time of 50 min [42]. Chen et al. fabricated bio-oil via the liquefaction of corn stover, and identified the optimal process conditions to be a reaction temperature of 220 °C, a NaOH concentration of 25 wt%, and a holding time of 60 min. The yield trend predicted by a convolutional neural network (CNN) was in excellent agreement with the experimental results [43].

To further elucidate the technical differences and parametric characteristics of bio-oil production from diverse agricultural and forestry wastes, Table 2 collates the process conditions and optimization outcomes from representative peer-reviewed studies. This collation enables a systematic analysis of the variation patterns in product yield and application performance across different feedstocks and process routes.

### 3.3. Algae Oil

For asphalt rejuvenators derived from algal biomass, Nor-Insyirah et al. [48] and Kathirvel et al. [49] have successively developed two technical routes: hydrothermal liquefaction (HTL) and microwave-assisted HTL. Such processes enable the direct treatment of high-moisture algal feedstocks without pre-drying, achieving an energy consumption reduction of over 30%. Under subcritical water conditions of 250–350 °C and 4–20 MPa, these processes enable the directional production of bio-oil-rich products, which can replenish the light aromatic fractions lost during asphalt aging. Chaiwong et al. [50,51] achieved the degradation and conversion of Spirulina feedstock without complex catalytic systems, realizing effective conversion solely through the regulation of reaction temperature. The resulting bio-oil exhibits excellent compatibility with asphalt binders and can be directly applied for the rejuvenation of aged asphalt without secondary treatment. Szymon et al. [52] developed a rejuvenator with a three-dimensional (3D) network structure via the etherification crosslinking reaction between sodium alginate and epichlorohydrin. This rejuvenator delivers optimal performance at a dosage of approximately 2.5% by mass of asphalt binder and can be directly blended with reclaimed asphalt pavement (RAP) without high-temperature processing equipment to soften and restore the properties of aged asphalt. Abraham et al. [53] adopted a fast pyrolysis route for marine microalgae, where excessive carbonization was inhibited via a high heating rate in the range of 400–650 °C, achieving a bio-oil yield of 35–50%. The produced bio-oil has a significantly lower sulfur content than fossil-based (petroleum-derived) rejuvenators; after incorporation into asphalt, it can markedly mitigate asphaltene agglomeration and improve the flowability and ductility of the binder. Overall, algal feedstocks are characterized by abundant oxygen-containing functional groups, active thermal degradation behavior, and excellent compatibility with asphalt binders, providing a promising and advantageous feedstock pathway for the development of rejuvenated asphalt systems.

### 3.4. The Influence of Preparation Process on Chemical Composition and Stability

Bio-oils derived from diverse feedstocks, including vegetable oils, lignin-derived oils, straw-derived oils, algal oils, and cassava-derived oils, exhibit consistent patterns in the regulation of chemical composition and the modulation of storage stability via their preparation and purification processes.

In the preparation stage, the enrichment of target products is typically achieved by tuning reaction temperatures and process conditions, including pyrolysis at 450–600 °C, hydrothermal liquefaction at 250–350 °C, and transesterification at 55–65 °C. Meanwhile, alkaline catalysts (e.g., NaOH), heterogeneous catalysts, and co-solvents are employed to promote the formation of target components such as fatty acid methyl esters (FAME), phenols, and hydrocarbons, while reducing the proportion of oxygenated, nitrogenous, and sulfur-containing impurities.

The subsequent purification step further removes residual reaction solids and undesired impurity components; for instance, the phospholipid content can be reduced from 138.8 ppm to 4.35 ppm, thereby minimizing interference in subsequent reactions and mitigating the risk of performance degradation during storage. With respect to storage stability, optimized process conditions can avoid excessive cracking of organic components and unwanted saponification side reactions, and the risks of oxidative degradation and polymerization are effectively mitigated via process strategies including short residence times, mild enzymatic catalysis, and easily separable catalytic systems. The reduced impurity content after purification also inhibits the increase in acid value and the formation of resins, while improving the reliability of long-term storage and continuous industrial operation. Relevant studies have demonstrated that bio-oils produced via optimized preparation and purification processes can maintain stable performance under long-term operating conditions. For example, a palm-based biogas production system achieved stable continuous operation for 7920 h per year without performance anomalies, and purified rapeseed-derived bio-oil exhibited no phase separation after 30 days of ambient storage [54].

These consistent patterns confirm a definitive correlation between preparation/purification processes, chemical composition, and storage stability of bio-oils. Rational selection of preparation and purification routes is therefore critical to guaranteeing the functional performance and engineering applicability of bio-oil-based asphalt rejuvenators.

**Figure 3:**
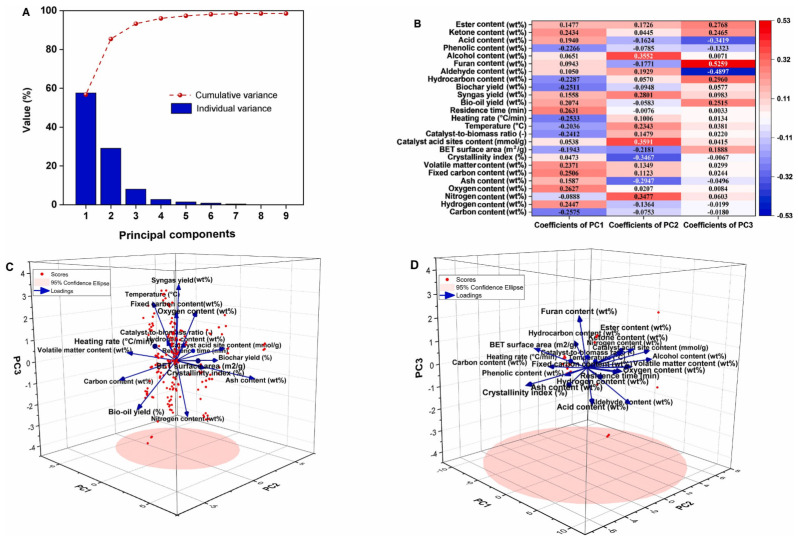
The results of principal component analysis for database reconstruction include (**A**) variance of each component, (**B**) correlation between each descriptor and the first three principal components, (**C**) loading plot of input parameters on product distribution, and (**D**) loading plot of input variables on bio-oil components [55].

Furthermore, Chen et al. verified the reliability of a data imputation method by processing a reconstructed raw dataset through Principal Component Analysis (PCA). As shown in Figure 3A, the cumulative variance contribution rate of the first three principal components exceeds 90%, enabling the core information of the dataset to be captured efficiently. Figure 3B illustrates that the first principal component is closely associated with biomass feedstock composition (elemental and compositional analysis), while the second principal component correlates with process operating conditions and catalyst properties. This finding highlights the critical importance of variable selection for optimizing the efficiency of in situ catalytic pyrolysis of biomass. Analysis of variable correlations via loading plots (Figure 3C,D) yielded the following key findings: bio-oil yield is positively correlated with the volatile matter content of the biomass feedstock and negatively correlated with its ash content. Specifically, high volatile matter content promotes higher bio-oil yields, while elevated ash content tends to induce problems such as catalyst deactivation via poisoning; the catalyst-to-biomass mass ratio is positively correlated with the hydrocarbon content of the resulting bio-oil, and increasing this ratio enhances the deoxygenation degree of the bio-oil while reducing the content of oxygenated compounds.

These findings elucidate the intrinsic correlations between these key variables, providing critical theoretical support for the directional optimization of biomass catalytic pyrolysis processes and the targeted production of bio-oil products with designed properties [55].

**Figure 4:**
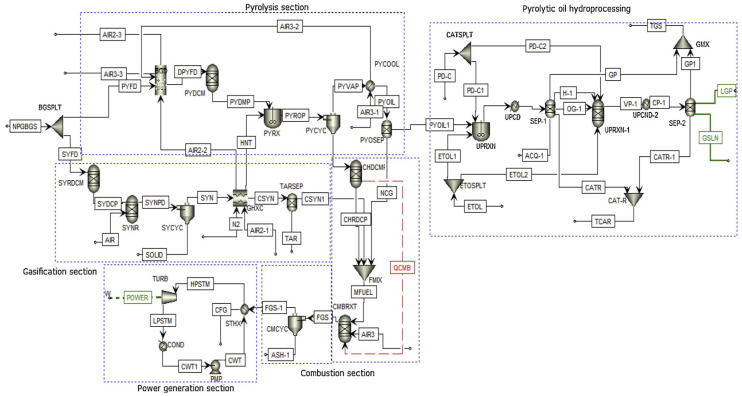
Process flow for biomass refining through thermochemical conversion [54].

Mohammed’s process design for integrated biomass refining via thermochemical conversion was implemented in ASPENPLUS^®^ V10 software. The process flow diagram adopts Non-Random Two-Liquid (NRTL) thermodynamics and performance data, with its core pathway as follows: Biomass is cracked in the pyrolysis section to produce crude bio-oil and combustible gasses; the gasses are utilized for power generation to achieve energy recycling; the crude bio-oil then enters refining sections such as hydrotreating for deoxygenation and stabilization upgrading, ultimately yielding a high-quality and high-stability bio-oil product, as illustrated in Figure 4 [54]. This figure shows a complete process system that integrates pyrolysis, gasification, combustion, power generation, and pyrolysis oil hydrogenation refining, which can achieve multi generation conversion of raw materials and comprehensive utilization of energy.

To summarize, biomass resources such as vegetable oils, lignin derivatives, and algal oils provide a diverse feedstock foundation for asphalt rejuvenators. Among these, the vegetable oil system has achieved high conversion rates and mature technical routes through processes including transesterification, supercritical methanol treatment, and heterogeneous catalysis. Lignin and agricultural/forestry wastes can yield high bio-oil production rates via pyrolysis, liquefaction, or catalytic cracking, with optimized process parameters derived from model-based optimization. Meanwhile, the algal oil system has demonstrated promising application potential due to its inherent advantages—adapting to high-moisture feedstocks, eliminating the need for pre-drying, and exhibiting excellent compatibility with asphalt.

Despite the progress made in current research regarding yield enhancement, impurity control, and environmental benefits, there remains a lack of validation at the actual engineering scale. Moving forward, efforts should be focused on strengthening the precise regulation of reaction processes, establishing a unified evaluation system and on-site validation mechanism, and promoting the large-scale, long-term reliable application of bio-based rejuvenators in road engineering.

## 4. Chemical Composition and Physicochemical Properties of Biomass-Based Rejuvenators

Binders have progressively advanced from macro-performance characterization to molecular-scale mechanistic analysis, with their capacity to restore asphalt performance being intrinsically linked to their complex chemical compositions and tailored molecular structures. Bio-based rejuvenators typically contain multiple classes of chemically active components concurrently, and exhibit a distinct amphiphilic molecular architecture characterized by the coexistence of polar and non-polar segments. This unique structural feature enables them to engage in multi-dimensional interactions with the saturates, aromatics, resins, and asphaltenes (SARA fractions) in asphalt binders, thereby driving key processes including the depolymerization and dispersion of asphaltene aggregates, as well as the reconstruction of the asphalt colloidal structure. Accordingly, the systematic elucidation of the primary active components, molecular structural characteristics of these rejuvenators, and their interaction potential with asphalt SARA fractions is fundamental to unraveling their underlying rejuvenation mechanisms and establishing tailorable, designable bio-based asphalt rejuvenation systems.

### 4.1. Main Active Components

The active components of bio-based rejuvenators originate from the extraction and thermochemical conversion of biomass feedstocks. The targeted restoration of the performance of aged asphalt binders is achieved through the synergistic effects of these distinct components, whose core active substances can be broadly categorized into four primary classes: fatty acid esters, phenols, aldehydes and ketones, and terpenes.

Regarding the source and experimental verification of these active components, Li et al. characterized the chemical composition of corn stover-derived bio-oil via gas chromatography-mass spectrometry (GC-MS). The results indicated that the bio-oil is mainly composed of phenols, ketones, alkanes, and other organic compounds [56], which is in full agreement with the findings of Nizamuddin et al. from their investigation into the solvothermal liquefaction of corn stover [57]. Fatty acid esters are formed by the esterification of long-chain fatty acids with polyols. The ester groups in their molecular structure enable excellent compatibility and interfacial interactions with the hardened resins and asphaltenes in aged asphalt binders; simultaneously, the long-chain alkyl groups can fill the intermolecular voids in the asphalt matrix, thereby mitigating the brittleness and stiffness of the binder [58,59].

Phenolic molecules contain phenolic hydroxyl groups and aromatic rings, endowing them with inherent antioxidant properties and polar adsorption capacity. During the rejuvenation process, phenolic compounds can scavenge free radicals through their phenolic hydroxyl groups and inhibit the polymerization of asphaltenes, thus retarding the progressive oxidative aging of the asphalt binder [60]. Aldehydes and ketones contain aldehyde or ketone functional groups, which are characterized by high polarity and chemical reactivity. Despite their relatively low content in bio-oils, they can regulate the aggregation state of asphaltenes and reduce their molecular weight via esterification and condensation reactions with asphaltenes in the asphalt binder [61,62]. Meanwhile, the high polarity of aldehydes and ketones can improve the interfacial compatibility between the rejuvenator and aged asphalt binder, preventing phase separation during storage. Meng et al. prepared asphalt modifiers using different aldehydes, which were converted into macromolecular resinous substances. This modification not only reduced volatile organic compound (VOC) emissions but also enhanced the high-temperature stability and moisture stability of the asphalt binder [63]; consistent conclusions were reported in the study by Cao et al. [64]. Furthermore, Li et al. developed asphalt rejuvenators using ketone-rich bio-oil, which achieved an enhanced performance restoration effect on aged asphalt binders [65].

Terpenes are a class of isoprene-based compounds, where isoprene (a conjugated diene) exhibits thermal reaction behavior consistent with the typical characteristics of diene compounds. At relatively low temperatures, the dominant reaction of isoprene is polymerization, generating higher-order terpene compounds. As the temperature increases, isoprene undergoes decomposition reactions to form ethylene, propylene, butadiene, and other low-molecular-weight hydrocarbons; a further increase in temperature leads to the formation of aromatic compounds. These terpene-derived compounds exhibit low viscosity and high volatility [66], whose core function is to reduce the viscosity of aged asphalt binders. The non-polar long chains of terpenes can intercalate between asphalt molecular chains, disrupting the agglomerated structure of asphaltene aggregates. Meanwhile, the unsaturated double bonds can undergo copolymerization with unsaturated components in the asphalt binder, thus improving the elastic recovery performance of rejuvenated asphalt [67].

### 4.2. Molecular Structural Features

From the perspective of molecular structure, the molecular architecture of bio-based asphalt rejuvenators is dominated by an amphiphilic structure characterized as “polar groups–non-polar segments–polar groups”. Polar groups are mostly located at the ends or side chains of molecular chains, responsible for binding to the highly polar resins and asphaltenes in aged asphalt; while non-polar segments serve as the main molecular backbone, which can be compatible with saturates and aromatics in asphalt, filling molecular gaps and improving molecular packing. For instance, fatty acid ester molecules exhibit a “linear amphiphilic structure”, with a polar ester group at one end and a non-polar C16–C18 alkyl group at the other. This linear structure endows them with excellent dispersibility in the asphalt matrix [68]. In contrast, phenolic molecules possess a “cyclic amphiphilic structure”, where the aromatic ring acts as the non-polar core and phenolic hydroxyl groups as the polar end groups. This structure enables the rejuvenator to not only form chemical bonds with the polar components of aged asphalt but also achieve physical compatibility with non-polar components, thereby avoiding interfacial separation [69]. Xu et al. established molecular dynamics (MD) models to analyze the diffusion mechanisms and influencing factors of different types of molecules and further validated the regulatory laws of factors (including temperature and the degree of asphalt aging) on molecular diffusion behavior [70].

### 4.3. Potential for Interaction with SARA Components of Aged Asphalt

For saturates, the non-polar components in bio-based rejuvenators can achieve “like-dissolves-like” compatibility with saturates [71]. They fill the molecular gaps of saturates through physical mixing to enhance the fluidity of saturates; simultaneously, the unsaturated components in the rejuvenator can undergo mild copolymerization with saturates, increasing the molecular weight of saturates, preventing their excessive volatilization at high temperatures, and improving the high-temperature stability of rejuvenated asphalt [72].

For aromatics, the aromatic ring structures in bio-based rejuvenators can form stable bonds with aromatics through π-π stacking interactions, thereby increasing the relative content of aromatics. On one hand, phenols and aldehydes/ketones in the rejuvenator can degrade asphaltenes, converting part of the asphaltenes into aromatics and increasing the aromatic content by 5–10% [73]; on the other hand, the aromatic components of the rejuvenator can directly supplement the aromatics lost in aged asphalt, improving the colloidal structure of asphalt and transforming it from a “sol–gel” type to a “sol” type, which effectively reduces asphalt viscosity [74,75,76]. Chen et al. validated this viewpoint regarding the replenishment of aromatics and improvement of colloidal structure by rejuvenators through experiments using waste cooking oil (WCO) and waste wood oil (WWO).

For resins, the polar components in bio-based rejuvenators can form strong interactions with resins. First, hydrogen bonding enhances the molecular stability of resins and inhibits the deterioration process of resins converting into asphaltenes [77]; second, polar adsorption forms a protective layer on the surface of resin molecules, hindering the polymerization reaction between resin molecules. Li et al. demonstrated that lignin-based phenolic rejuvenators can increase the dispersibility of resins, improving the deformation resistance and fatigue performance of asphalt [78].

For asphaltenes, the interaction between bio-based rejuvenators and asphaltenes is the core of the rejuvenation process, primarily achieved through a “depolymerization-dispersion” mechanism: (1) Chemical depolymerization: Aldehydes/ketones and phenols in the rejuvenator can attack the macromolecular chains of asphaltenes through electrophilic substitution reactions, cleaving their C-C and C-O bonds, decomposing asphaltenes into small-molecular aromatics and resins, and reducing the relative content of asphaltenes. This conclusion has been confirmed by Zheng et al., who compared the decomposition effect by fabricating nanoclusters and re-decomposing them with substances such as WCO. (2) Physical dispersion: The amphiphilic components in the rejuvenator can adsorb onto the surface of asphaltene particles—binding to asphaltenes via polar groups while the non-polar groups are compatible with aromatics and saturates outwardly—forming a stable dispersion system and preventing the re-agglomeration of asphaltene particles [79].

**Figure 5:**
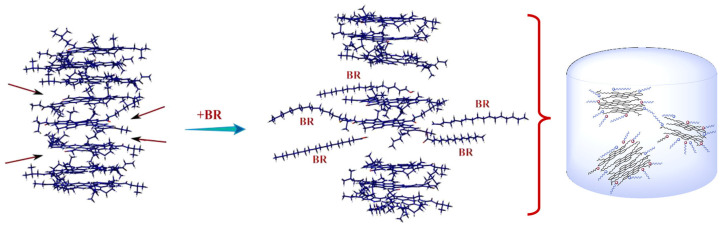
Mechanism of biomass rejuvenator decomposing aged asphalt polymer [80].

**Figure 6:**
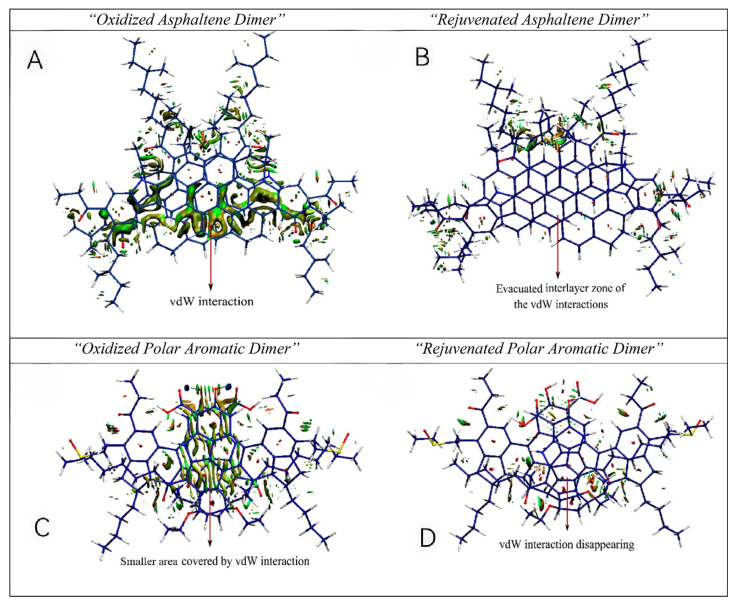
Analysis of non-covalent interactions between molecules of asphaltene monomers in (**A**,**C**) oxidized dimers and (**B**,**D**) regenerated dimers (top view) [80].

Furthermore, the Pahlavan research team simulated the interference of rejuvenators on the electron distribution in aged asphalt, as illustrated in Figure 5. Rejuvenators can penetrate into the molecular gaps between two interacting asphaltene molecules in the polymer and perturb the electron cloud formed during asphalt aging through polar electron absorption and CH-π interactions between hydrocarbon tails and the polycyclic aromatic hydrocarbon (PAH) cores at the ends of asphaltenes. As shown in Figure 6A,C, there are no significant attractive hydrogen bonds or repulsive steric forces in the oxidized dimers. Van der Waals forces are displayed as extended green surfaces between fragments (green interfaces in Figure 6). The van der Waals forces and electrostatic interactions between rejuvenated oxidized asphaltene fragments (Figure 6B) and rejuvenated non-asphaltene components (Figure 6D) almost disappear [80,81].

Taken together, existing studies have demonstrated that the active components (i.e., fatty acid esters, phenols, aldehydes and ketones, and terpenes) in bio-based rejuvenators exert synergistic effects on the SARA fractions of asphalt binders via their inherent amphiphilic structure, which integrates both polar and non-polar moieties. These components restore the colloidal stability and viscoelastic properties of aged asphalt binders through multiple complementary mechanisms, including asphaltene depolymerization, dispersion of asphaltene aggregates, and reconstruction of the asphalt colloidal network structure.

However, at the current research stage, there remains a critical lack of accurate quantitative characterization of the contribution of key active functional groups and their synergistic interactions. Furthermore, investigations into the influence of complex impurities and inherently unstable components in crude bio-oil on the long-term service performance of rejuvenated asphalt binders are still far from comprehensive and in-depth. Future research should therefore focus on the development of quantitative contribution models for active components and the establishment of functional molecular design strategies, combined with the integration of multi-scale numerical simulation and cyclic aging evaluation methods. This will ultimately promote the paradigm shift in bio-based rejuvenator research and development from an experience-dependent empirical framework to a mechanism-driven scientific system.

## 5. Asphalt Aging Mechanism and the Necessity of Regeneration

### 5.1. Component Changes During Asphalt Aging Process

Asphalt is a type of viscoelastic semi-solid or solid material primarily composed of high-molecular-weight hydrocarbons and their derivatives. From a chemical composition perspective, it is core composed of asphaltenes, resins, aromatics, and saturates [82]. The relative content of each fraction and their intermolecular interactions collectively determine the macroscopic properties of asphalt. Changes in the weight distribution of fractions are key indicators for characterizing the evolution of its chemical components [83,84,85].

**Figure 7:**
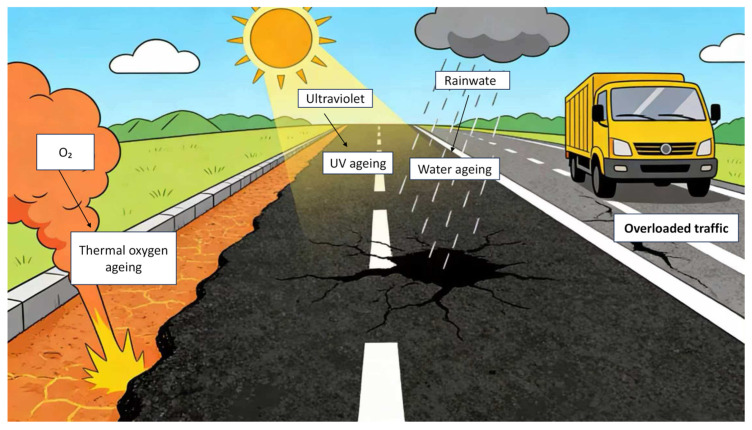
Schematic diagram of asphalt aging factors.

Asphalt aging occurs when asphalt is exposed to environmental factors such as heat, oxygen, sunlight, and water for extended periods throughout the entire process from refineries to storage, transportation, construction, and service. Such exposure induces a series of physical and chemical changes, including volatilization, oxidation, decomposition, and polymerization. These changes alter the properties of asphalt, leading to modifications in its internal molecular structure and chemical composition. This irreversible process renders asphalt dry, brittle, and stiff, with oxidation-induced hardening being the primary cause of asphalt aging, as illustrated in Figure 7.

Qu et al. employed the Corbett fractionation method to separate the components of asphalt with different aging degrees. The experimental results clearly showed that the aromatic content decreased, while the asphaltene content exhibited a significant increasing trend [86]. Ma et al. further verified through component separation and composition comparison of different grades of asphalt under the same conditions that the essence of asphalt aging is a process in which light fractions gradually convert to heavy fractions driven by oxidation reactions.

**Figure 8:**
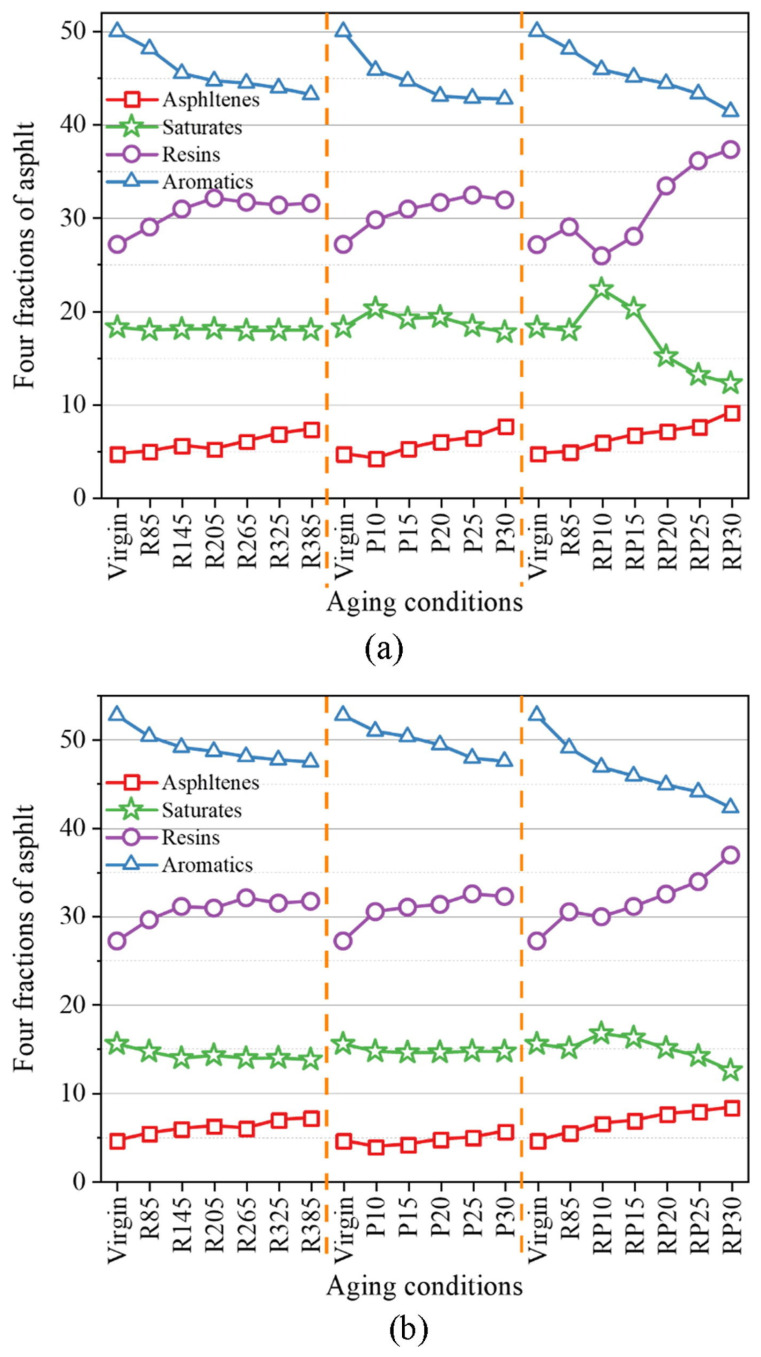
Variations in asphalt four-component fractions under different aging conditions. (**a**) Four-component distribution of neat asphalt during thermal aging and photo-thermal aging; (**b**) Four-component distribution of rejuvenated asphalt under the same aging regimes [87].

Ma et al. also investigated the compositional changes in asphalt during aging under different conditions, as shown in Figure 8. During asphalt aging, the asphaltene content increases significantly, the change in resin content varies with asphalt type, and both saturate and aromatic contents decrease. The reduction in saturates stems from thermal volatilization and oxidative polymerization into resins or asphaltenes, while the easy oxidation of branched alkyl groups in aromatics into carboxyl groups results in an increase in the hydrogen-carbon (H/C) ratio of asphalt. There is a dynamic two-way transformation between resins, saturates, and aromatics—specifically, the latter two polymerize to form resins, which can in turn convert to asphaltenes. The compositional changes vary among different asphalts: the changes in M1 and M2 asphalts are relatively slight, while those of M3 are more pronounced under cyclic conditions [87].

**Figure 9:**
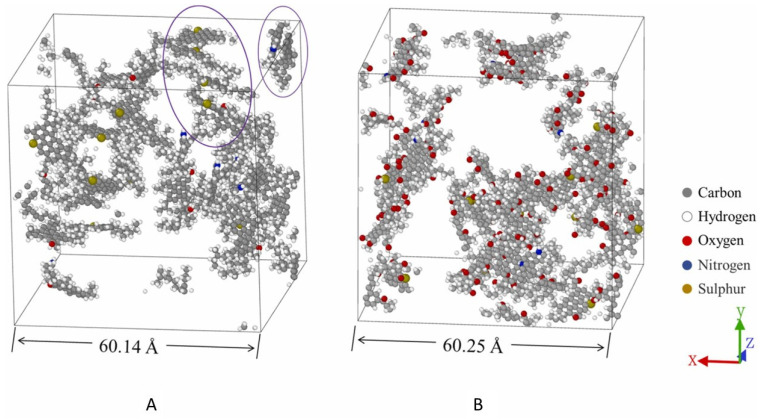
The aggregation state of asphalt in both original (**A**) and aged asphalt (**B**) [89].

Furthermore, Ren et al. further elucidated the influence mechanism of asphaltene content changes on the microstructure through comparative tests of short-term and long-term aging. With the increase in asphaltene content, the relative distance between asphaltene molecules decreases, intermolecular interactions are enhanced, and nanoparticle agglomeration is aggravated [88]. Han et al. used molecular dynamics simulation, as illustrated in Figure 9, to show the molecular morphology of asphaltenes (with other components removed) in pristine and aged asphalt models after sufficient relaxation at 24.85 °C (298 K). The asphaltene molecules did not agglomerate but existed in a dispersed state and aggregated in a randomly oriented manner; only a small number of parallel-aligned molecules were observed in Figure 9A (marked by circles), and the dispersion heterogeneity confirms their aggregation tendency. Aging promotes this aggregation tendency: as shown in Figure 9B, oxidized asphaltene molecules exhibit a higher degree of agglomeration than pristine asphaltene molecules [89].

These studies collectively demonstrate that the essence of asphalt aging is the gradual evolution of the system from a monodispersed state to large-sized aggregates. The colloidal structure transforms from a state where asphaltenes are uniformly dispersed with soft performance to a state where asphaltenes agglomerate with hard and brittle performance. This thus leads to increased viscosity and decreased fluidity of the entire system, and under certain conditions, cracking occurs due to internal stresses generated by colloidal agglomeration.

### 5.2. Evolution of Molecular Structure During Asphalt Aging

**Figure 10:**
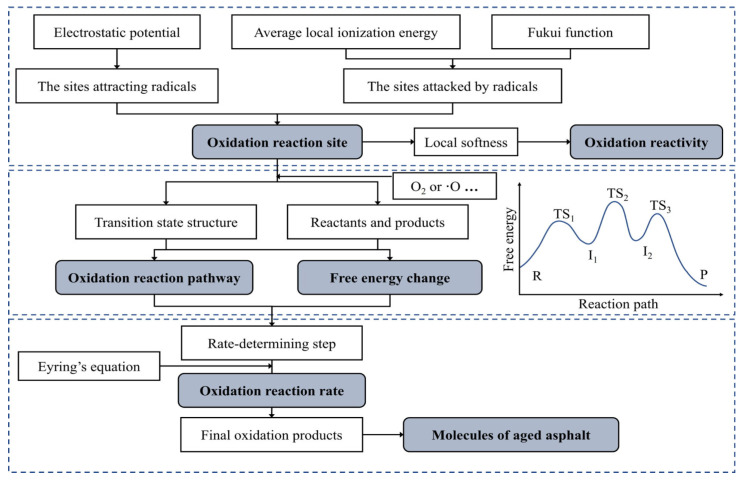
Schematic of oxidation reaction mechanism and molecular model of asphalt aging [94].

**Figure 11:**
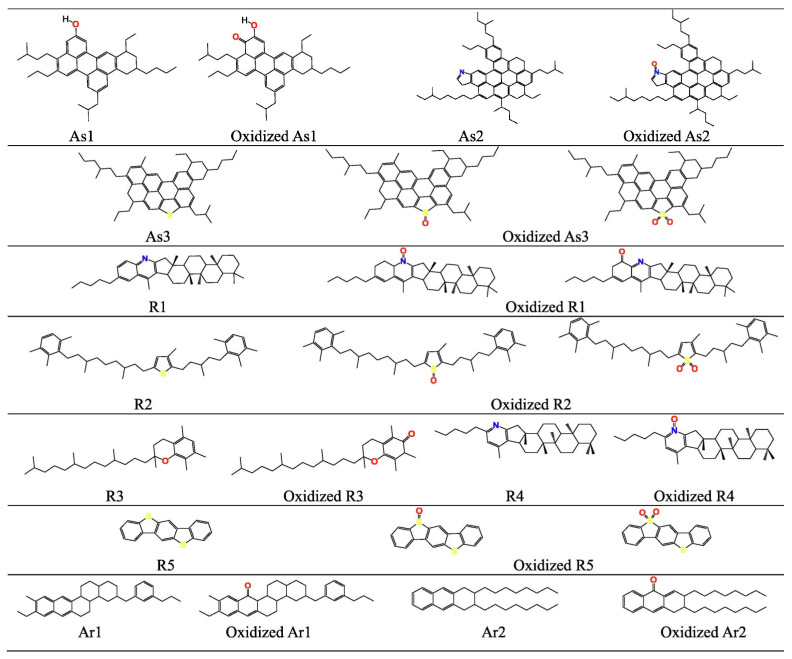
Asphalt molecule oxidation model [94].

Asphalt aging is an irreversible evolutionary process driven by the combined effects of oxidation reactions, polymerization reactions, loss of aromatic components, and accumulation of polar groups. Among these, oxidation reactions are generally recognized as the fundamental and crucial link, running through the entire aging process. Both thermo-oxidative aging and photo-oxidative aging are dominated by oxidation, and their mechanisms can be attributed to free radical chain reactions induced by thermal energy, ultraviolet (UV) radiation, or catalytic metal ions in aggregates [90]. Chen et al. observed the ultraviolet aging gradient using customized fluorescence microscopy, providing direct experimental evidence for the “diffusion aging model” [91,92]. The Ma research team analyzed pressure aging vessel (PAV) asphalt via Fourier transform infrared spectroscopy (FTIR) and found that the characteristic peaks of carbonyl (C=O) and sulfoxide (S=O) groups were significantly enhanced [93]. Liu’s study indicated that rolling thin film oven test (RTFOT) aging tends to follow a dehydrogenative oxidation mechanism [94]. The intermediate products generated by oxidation reactions further promote polymerization: aromatic components gradually form resins through addition polymerization, and resins then convert into asphaltenes via condensation polymerization [95]. Liu et al. conducted an in-depth analysis of the oxidation mechanism using quantum mechanical methods, as shown in Figure 10, involving three steps: predicting oxidation sites and reactivity; predicting reaction pathways and free energy changes; and calculating reaction rates. Ultimately, the strategies for improving asphalt aging resistance and the molecular model of aged asphalt were identified. The constructed oxidation molecular model is illustrated in Figure 11 [94], where each asphalt molecule contains an average of one oxygen-containing group—consistent with the experimental findings of Peterson et al. Pan et al.’s research demonstrated that the accumulation of oxidative functional groups enhances intermolecular binding forces and bulk modulus, which is the direct cause of the hardening of oxidized asphalt [96]. Polymerization reactions can be regarded as a secondary process of oxidation. By promoting molecular chain growth, they gradually convert small-molecular aromatics and medium-molecular resins into macromolecular resins and higher-molecular-weight asphaltenes, serving as the primary driving force for the heavy fraction evolution of SARA components [97,98,99,100]. The continuous accumulation of polar groups stems from the decomposition of hydroperoxides and the oxidation of sulfur-containing compounds. Computational chemistry studies have pointed out that asphaltene molecules exhibit high polarizability, making them more prone to oxidative functionalization. The newly formed polar groups significantly strengthen intermolecular forces, thereby promoting the aggregation of asphaltene particles into flocculent structures [101,102].

### 5.3. Degradation of Performance Due to Asphalt Aging

The performance degradation of asphalt binders induced by aging is primarily manifested in the deterioration of rheological properties, an elevated risk of low-temperature brittle cracking, and the loss of viscoelastic equilibrium. These detrimental changes are intrinsically linked to the oxidative polymerization of asphalt components and the flocculation behavior of the asphalt colloidal structure. At present, the quantitative evaluation of asphalt aging degree is mainly based on key rheological indicators, including viscosity, softening point, complex shear modulus (G*), and phase angle (δ) [103,104].

With respect to the evolution of rheological properties, aging induces a marked increase in the dynamic viscosity and complex shear modulus of asphalt binders, accompanied by a significant decrease in the phase angle. Studies on rolling thin film oven test (RTFOT)-aged and pressure aging vessel (PAV)-aged asphalt binders conducted by Zhao et al. [105], Zhang et al. [106], and Wang et al. [107] have demonstrated that the dynamic viscosity of aged asphalt at 60 °C can reach 3–5 times that of unaged virgin asphalt, while the phase angle decreases from approximately 45° to below 30°. This transition indicates that the asphalt binder shifts from a viscosity-dominated viscoelastic equilibrium state to an elasticity-dominated hard and brittle state, with a substantial reduction in its deformability and fluidity.

Furthermore, Bending Beam Rheometer (BBR) test results reported by Zhou et al. [108] and Soenen et al. have confirmed that under low-temperature conditions, the creep stiffness of aged asphalt binders is 2–3 times higher than that of unaged asphalt, with a significant decrease in the creep rate (m-value). This finding reveals that the aged material is unable to dissipate thermal stress effectively via viscous deformation, thereby drastically increasing the probability of pavement cracking in low-temperature environments.

### 5.4. Rejuvenator’s Restoration of Asphalt Performance

Rejuvenators represent the core functional materials for the performance restoration of aged asphalt binders, whose fundamental role is to regulate the chemical composition and microstructure of asphalt binders, thereby reversing the performance degradation trend induced by oxidative aging processes. From the perspective of chemical composition regulation, rejuvenators first remediate the compositional imbalance of aged asphalt binders through component supplementation and dilution effects. On one hand, they replenish the light fractions lost via volatilization and oxidation during the aging process, effectively correcting the proportional imbalance among the saturates, aromatics, resins, and asphaltenes (SARA) fractions. On the other hand, the active components in rejuvenators can optimize the dispersion state of heavy fractions (resins and asphaltenes) and mitigate their agglomeration tendency through solvation and dilution effects. Hu et al. employed hydrothermally treated corn stover as a rejuvenator and confirmed that this material can significantly inhibit the formation of asphaltene clusters [109]. Ding et al. further noted that bio-oil produced via the hydrothermal liquefaction of lignocellulose is rich in hydroxyl, ester, and aromatic groups, which endows it with excellent compositional regulation potential and lays the chemical foundation for subsequent performance restoration [110].

At the microstructural level, a key mechanism for asphalt performance improvement is that rejuvenators can drive the transition of the colloidal structure of aged asphalt binders from a disequilibrium state to a thermodynamically stable state. Ke et al. elucidated the rejuvenation effect of waste soybean oil (WSO) on aged asphalt binders through a combination of laboratory tests and molecular dynamics simulations. They demonstrated that WSO can effectively inhibit the evolution of the asphalt colloid towards a “gel-type” hard and brittle structure and promote its regression to a more stable “sol–gel equilibrium-type” structure, which lays the microstructural foundation for the recovery of macroscopic performance [111]. The optimization of this colloidal structure is further reflected in the improvement of macroscopic engineering properties: it effectively alleviates the rheological hardening of aged asphalt binders, reduces the risk of low-temperature brittle cracking, restores the balance of viscoelastic characteristics, and enhances the fatigue resistance and low-temperature deformation resistance of the asphalt binder. Research by Mohammad et al. found that the critical cracking temperature of bio-asphalt mixtures incorporating pine bio-oil is as low as −22 °C, which significantly outperforms that of the virgin base asphalt binder, with a substantial improvement in low-temperature crack resistance [112].

**Figure 12:**
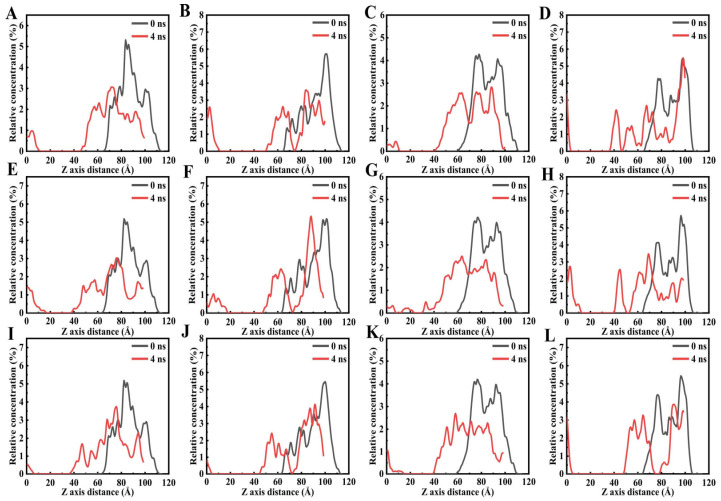
Relative concentration distribution of different components in aged asphalt [113].

**Figure 13:**
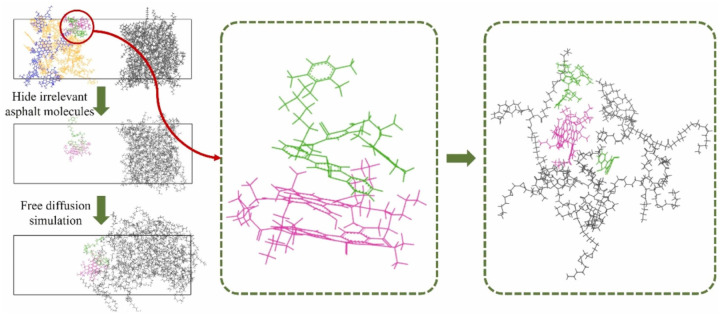
Declustering phenomenon in diffusion models [113].

Furthermore, rejuvenators can optimize the interfacial interaction between asphalt binders and mineral aggregates, thereby enhancing the overall performance stability of recycled asphalt mixtures. However, it should be noted that the rejuvenation efficacy is governed by multiple factors, including the inherent chemical composition of the rejuvenator, the aging degradation degree of the target asphalt binder, and the dosage of the rejuvenator. As illustrated in Figure 12, Chen et al. clearly demonstrated the influence of rejuvenators on each SARA fraction of asphalt binders, which confirmed that heavy fractions tend to agglomerate after oxidative aging. Meanwhile, following the diffusion of the rejuvenator, the correlation at the characteristic peak positions of asphaltenes and other heavy components decreases significantly, indicating that asphaltene agglomeration is effectively alleviated. The corresponding molecular simulation results are presented in Figure 13 [113]. Therefore, it is essential to establish a scientific compatibility evaluation system between rejuvenators and aged asphalt binders through systematic research, to achieve the optimal performance restoration effect [114,115,116].

To summarize, asphalt aging is characterized by the loss of light fractions, accumulation of polar structures, and gradual agglomeration of asphaltenes. The colloidal system evolves from a dispersed state to a gel state, thereby inducing performance degradation such as rheological hardening, low-temperature embrittlement, and disruption of viscoelastic equilibrium. Rejuvenators effectively improve rheological properties and low-temperature toughness, as well as enhance fatigue resistance and interfacial adhesion performance, by supplementing light fractions, weakening asphaltene agglomeration, and restoring the colloidal structure.

However, there remain research gaps in the molecular regulatory mechanisms, diffusion kinetics, and long-term durability during the aging-rejuvenation process. Current evaluation systems are mostly confined to laboratory characterization, with insufficient evidence for long-term engineering service. Future research is suggested to strengthen studies on multi-scale structure-property relationships, develop targeted precision rejuvenator systems based on directional functional groups, and establish a long-term performance evaluation and on-site validation system under multi-environmental conditions. This aims to promote the deepening of the rejuvenation mechanism from “component compensation” to “structural reconstruction,” ultimately achieving the goals of green recycling and long-term service of asphalt materials.

## 6. Performance Enhancement of Aged Asphalt by Biomass-Based Rejuvenator

### 6.1. Improvement of Rheological Properties

Rheological properties are the core characterization of the viscoelastic behavior of rejuvenated asphalt. Test results from a Dynamic Shear Rheometer (DSR) and Bending Beam Rheometer (BBR) are directly correlated with the high and low-temperature service performance of pavements. After regulation by rejuvenators, the complex shear modulus (G*), phase angle (δ), and rutting factor (G*/sinδ) of aged asphalt can achieve precise optimization. Specifically, in high-efficiency rejuvenation systems, the synchronous improvement of G* and G*/sinδ enhances high-temperature deformation resistance, while a moderate reduction in G*/sinδ can improve construction workability. In the low-temperature range, the stiffness modulus (S) and creep rate (m-value) are key indicators of crack resistance [117]. For instance, high-quality rejuvenators can significantly reduce S and increase the m-value, optimizing low-temperature stress relaxation capacity. Among these, the m-value improvement of some bio-based systems is more suitable for cold-region scenarios. Through the compounding of rejuvenators, equivalent matching of G*/sinδ with that of base asphalt can be achieved, while avoiding high-temperature critical temperature shift—providing support for the engineering-oriented regulation of rheological properties [118].

High and low-temperature performance, along with fatigue crack resistance, collectively determine the full-temperature-range durability of recycled asphalt pavements. In the high-temperature range, researchers have proposed that a reasonable increase in the G*/sinδ can directly inhibit permanent deformation. The complex shear modulus of some rejuvenation systems can be restored to the level of base asphalt, with no significant difference in high-temperature rutting resistance compared to the original material. In the low-temperature range, in addition to BBR indicators, the improvement of fracture toughness can also reduce the cracking risk. The low-temperature fracture toughness of high-quality composite rejuvenation systems can be increased by more than 40% [119,120]. Regarding fatigue crack resistance, Linear Amplitude Sweep (LAS) and Multiple Stress Creep Recovery (MSCR) tests have shown that high-performance rejuvenators can extend fatigue life (Nf), improve fatigue recovery rate (R), and reduce non-recoverable creep compliance (Jnr), thereby enhancing long-term fatigue resistance [121,122]. It should be noted that some rejuvenators exhibit a performance trade-off effect—for example, improving low-temperature crack resistance may weaken high-temperature rheological properties. Therefore, it is necessary to achieve a balance between the two through compounding.

### 6.2. Durability and Long-Term Anti-Aging

The actual service life of asphalt pavements is largely determined by their ability to resist long-term aging. In laboratory research, to rapidly and accurately evaluate this capability, it is necessary to rely on accelerated aging methods that simulate the actual degradation environment. Among these, the Pressure Aging Vessel (PAV) test, ultraviolet (UV) aging test, and thermal-humidity cycling test are the three most widely used core methods internationally [123,124].

**Figure 14:**
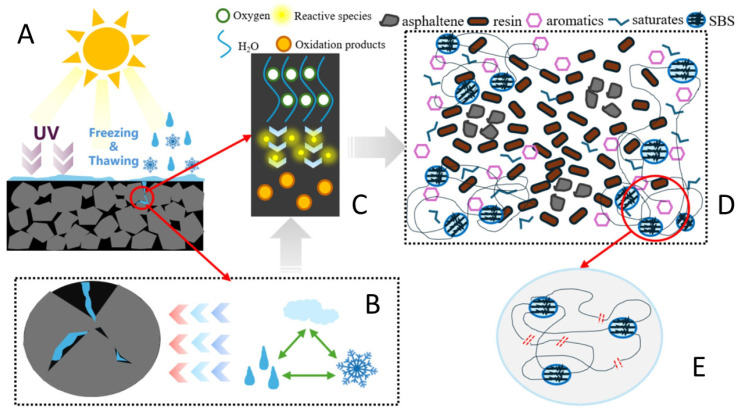
Aging mechanism of asphalt in UV-freeze–thaw environment [126].

The PAV test, by simulating high-temperature, high-humidity, and high-pressure oxygen environments, mainly reproduces the oxidative aging process of asphalt inside the pavement structure caused by long-term heat exposure and oxygen penetration. This type of aging leads to the hardening of the asphalt colloid and reduced adhesion, which is a key factor contributing to pavement cracking. The UV aging test focuses on the surface asphalt of pavements. By controlling the irradiation intensity of ultraviolet light with specific wavelengths, it simulates the photo-oxidative degradation effect of ultraviolet rays in sunlight on asphalt. Such aging of surface asphalt is often characterized by increased brittleness and degraded crack resistance, directly affecting pavement appearance and surface integrity. For example, Ju et al. found in their experiments that compared with pristine asphalt, RTFO-aged (Rolling Thin Film Oven-aged) and UV-aged asphalt exhibited new absorption peaks at 1030 cm^−1^ and 1648 cm^−1^, which correspond to sulfoxide and carbonyl functional groups, respectively. This indicates that significant oxidative reactions occurred in RTFO-aged and UV-aged asphalt. Compared with RTFO-aged asphalt, the carbonyl functional groups were more prominent, suggesting that the synergistic effect of UV radiation and oxygen results in a more pronounced aging degree of asphalt—UV aging accelerates asphalt degradation [125]. Meanwhile, He et al. demonstrated the aging mechanism of asphalt pavements under the coupled environment of UV radiation and freeze–thaw cycles, as illustrated in Figure 14 [126], elucidating the aging damage mechanism of modified asphalt under UV-freeze–thaw coupling. At the macroscopic level, pavement asphalt is simultaneously subjected to UV irradiation and freeze–thaw cycles; the latter induces asphalt microcracks through cyclic stresses caused by water infiltration, freezing expansion, and thawing disintegration. At the microscopic level, UV light activates reactive species, which undergo photo-oxidative reactions with oxygen and water to generate aging products. In the modified asphalt system where asphaltenes, resins, and other components were initially uniformly dispersed, molecular chain scission and crosslinking, as well as impaired dispersion of components, occur due to freeze–thaw internal stresses and photo-oxidative degradation, ultimately leading to the deterioration of macroscopic material properties.

Meanwhile, numerous researchers have proposed targeted test indicators and evaluation frameworks based on their respective research scenarios and technical routes, which has further enriched the theory and practice of asphalt performance evaluation [127,128,129]. Herein, this paper sorts out and summarizes the performance indicators of some oil-based rejuvenated asphalt, as shown in Table 3

Table 3 shows that vegetable oils, waste cooking oils, and algal oils exhibit outstanding performance in low-temperature crack resistance, while lignin derivatives, castor oil, and other similar materials demonstrate significant advantages in high-temperature rutting resistance—specifically by increasing the rutting factor (G*/sinδ) and reducing non-recoverable creep compliance (Jnr). However, pure vegetable oils such as waste cooking oils have a trade-off in high-temperature performance. In contrast, composite rejuvenators can achieve synergistic optimization of high and low-temperature performance and are suitable for engineering needs in different climatic regions.

### 6.3. Difference from Petroleum-Based Rejuvenator

Bio-based rejuvenators and petroleum-based rejuvenators share a similar core operational mechanism in restoring the performance of aged asphalt: both achieve the restoration of asphalt colloidal structure and macroscopic properties by supplementing the light fractions lost in aged asphalt and regulating the balance of SARA fractions. However, they exhibit significant differences in raw material sources, functional characteristics, and applicable scenarios.

Petroleum-based rejuvenators are derived from by-products of the petroleum refining process, with molecular compositions dominated by hydrocarbons. This inherent homology with asphalt enables them to rapidly penetrate into the interior of aged asphalt, efficiently accomplishing the rejuvenation process. In terms of performance advantages, petroleum-based rejuvenators are characterized by low cost, high rejuvenation efficiency, and excellent high-temperature stability, thus being widely applied in pavement recycling in high-temperature regions, emergency repair projects, and projects sensitive to cost control. Nevertheless, it should be noted that petroleum-based rejuvenators rely on non-renewable fossil resources, resulting in high carbon emissions during production and use. Additionally, their low-temperature crack resistance and interfacial adhesion with aggregates are relatively average, leading to limitations in low-temperature regions or scenarios requiring high interfacial stability [147].

In contrast, bio-based rejuvenators are derived from renewable biomass resources and have a high content of oxygen-containing compounds in their molecular structure. In addition to supplementing light fractions, they can also chemically modify asphalt through chemical reactions between functional groups, further enhancing performance. Their prominent advantages lie in low-temperature crack resistance, resistance to secondary aging, and environmental performance—characterized by low carbon emissions and environmental friendliness—thus making them more suitable for pavements in low-temperature regions, engineering projects with stringent environmental requirements, and high-durability pavements requiring long-term service.

The main limitations of current bio-based rejuvenators are as follows: limited by raw material processing and purification technologies, their cost is relatively high; the high-temperature stability of some types needs to be further improved through modification—for example, epoxy modification can be considered to enhance the thermal stability of ester groups [148]; moreover, the stability of a small number of biomass-derived rejuvenators during long-term storage or service still needs to be optimized.

## 7. Regeneration Mechanism of Biomass-Based Rejuvenator on Aged Asphalt

As research on bio-based rejuvenators gradually advances from macro-performance exploration to the level of microstructural and molecular mechanisms, their action mode for restoring the performance of aged asphalt has been recognized to exhibit multi-path and multi-scale coupling characteristics. Existing studies have shown that rejuvenators do not rely solely on dilution effects or functional group compensation but act synergistically through pathways such as molecular intercalation and segment softening, directional reactions of functional groups, and regulation of interfacial compatibility, thereby altering the aggregation morphology of asphaltenes, the stability of colloidal structures, and the balance of viscoelastic networks. From the supplementation and dispersion of light fractions to free radical scavenging and oxidation inhibition, and then to solubility parameter matching and interfacial energy reduction, these synergistic effects at the molecular and colloidal levels collectively drive asphalt to transition from a hard and brittle state to a system with both flexibility and toughness, resulting in significant improvements in rheological properties, low-temperature crack resistance, and aging retardation behavior.

Based on this, the action mechanism and microscopic evidence of bio-based rejuvenators are systematically elaborated below from four aspects: physical plasticization mechanism, chemical functional group complementarity, compatibility-driven colloidal structure reconstruction, and colloidal regulation mechanism.

### 7.1. Physical Dilution and Plasticizing Effect

The physical dilution and plasticization effects of bio-based rejuvenators on aged asphalt are mainly achieved by supplementing the light fractions lost during aging and weakening the internal forces of the colloidal structure. This process is corroborated by viscosity change behavior and microstructural evolution.

From the molecular perspective, for fatty acid ester-based rejuvenators represented by soybean oil and rapeseed oil-derived esters, their C16–C18 non-polar alkyl chains can insert into asphaltene aggregates through van der Waals forces, disturbing the hydrogen bonds and aromatic ring interactions within the aggregated regions. This leads to a significant reduction in the size of asphaltene clusters, thereby decreasing the system viscosity [149]. Xie et al. used a waste cooking oil–organophilic montmorillonite (WCO–OMMT) composite rejuvenator to improve the colloidal dispersion state of aged asphalt through light fraction supplementation and physical dilution [150]. The experimental results showed that at 135 °C, the insertion rate of alkyl chains when soybean oil fatty acid esters are mixed with aged asphalt is 0.8–1.2 nm/s, and the kinematic viscosity can be reduced from 300 to 400 mm^2^/s to 100–150 mm^2^/s after approximately 120 min. He et al. did not observe the formation of new functional groups when characterizing the rejuvenated asphalt, further indicating that the relevant system mainly functions through physical dilution [151].

**Figure 15:**
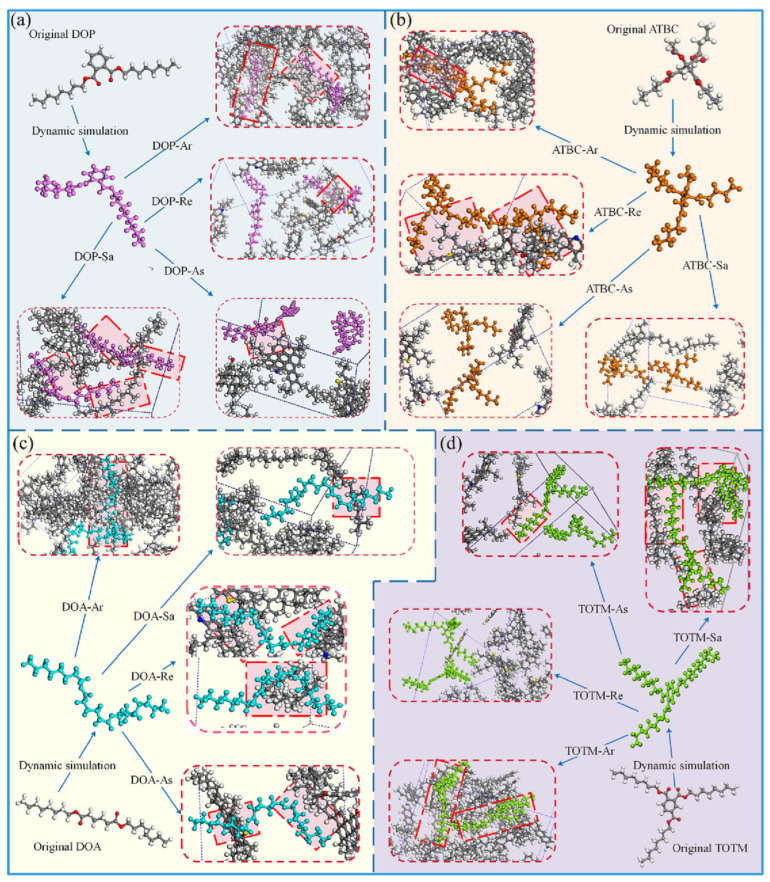
Spatial interactions between four plasticizers (DOP, ATBC, DOA, and TOTM) and asphalt components [149].

In terms of macroscopic performance, the low-temperature flexural strain of aged asphalt mixed with 8–12% fatty acid esters increases from less than 2000 με to 3000–4000 με. Zhang et al. observed via Atomic Force Microscopy (AFM) that the proportion of “bee-like” domains in the rejuvenated asphalt decreases from 35% to 15%, with a significant increase in soft-phase regions, confirming the contribution of the plasticization effect to flexibility improvement [152]. Notably, due to their low molecular weight and viscosity, terpene components can quickly fill the gaps between asphalt molecular chains and reduce intermolecular friction, with a dilution efficiency approximately 1.2–1.5 times higher than that of traditional petroleum-based rejuvenators [149,153,154]. Furthermore, low-boiling terpene components gradually volatilize during construction heating, which can avoid the high-temperature bleeding issue caused by excessively low viscosity. Gao et al. pointed out through molecular simulation studies that the matching of molecular polarity and spatial configuration between the rejuvenator and asphalt is a key factor affecting plasticization stability. If the system possesses strong non-bonding interactions and flexible segment deformation capacity, a more stable composite structure can be formed [155]. Figure 15 illustrates the spatial interactions between four types of plasticizers and asphalt components. It can be seen that linear molecular structures are more likely to adopt a bent spatial configuration to achieve charge balance and ultimately form a stable mixture. Aromatics contribute more than 35% to the total binding energy between asphalt and all plasticizers, with the highest contribution reaching 58%. Moreover, as the polarity of the plasticizer increases, the binding energy with the plasticizer also increases. This is mainly due to the relatively high aromatic content in asphalt; the more molecules interacting with the plasticizer, the greater the binding energy. This conclusion provides a theoretical basis for the molecular design of bio-based rejuvenators and the optimization of their physical action mechanisms.

### 7.2. Chemical Reaction and Functional Group Complementarity

**Figure 16:**
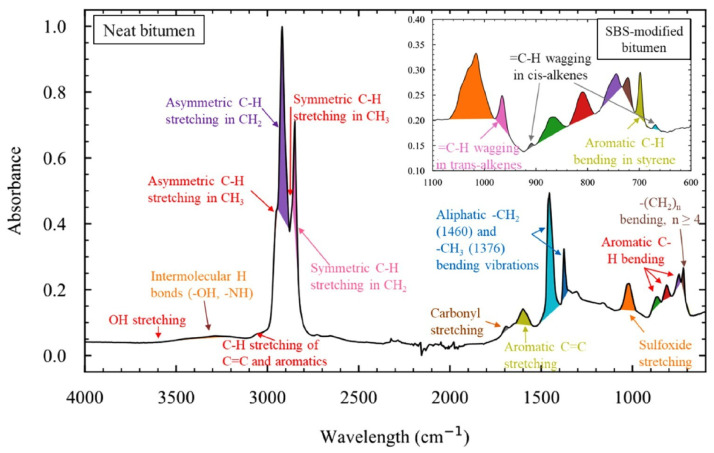
FTIR spectra of common base asphalt and SBS modified asphalt [159].

The chemical reactions between bio-based rejuvenators and aged asphalt are centered on the complementary interaction of their functional groups—specifically, active groups such as phenolic hydroxyl groups and carboxyl groups in rejuvenators undergo specific interactions with carbonyl and sulfoxide groups generated by oxidation in aged asphalt. This not only interrupts the oxidative chain reaction of asphalt but also restores the deteriorated colloidal structure, a mechanism corroborated by multiple experimental characterizations. Fourier Transform Infrared Spectroscopy (FT-IR) tests by Hu et al. revealed that compared with pristine asphalt, the intensity of characteristic peaks for carbonyl and sulfoxide groups in aged asphalt increased significantly; however, after adding bio-based rejuvenators, the intensity of these peaks decreased noticeably, indicating that rejuvenators effectively inhibit the accumulation of polar functional groups [156]. Ma et al. reached consistent conclusions in similar studies [93]. Additionally, Farideh et al. observed the microstructure of rejuvenated asphaltenes via High-Resolution Transmission Electron Microscopy (HRTEM) and speculated that low-molecular-weight compounds in bio-oil may exhibit superior solubility for oxidative aggregates in asphalt [157]. From the perspective of chemical reactions of specific components: The core role of phenolic components lies in antioxidation. The phenolic hydroxyl groups in their molecules can donate hydrogen atoms, which combine with peroxyl radicals generated during the oxidation of aged asphalt to form more stable phenoxyl radicals, thereby effectively interrupting the continuous progression of the oxidative chain reaction. Electron Paramagnetic Resonance (EPR) test data by Hamzeh et al. confirmed that after adding 5% lignin-based phenolic rejuvenator to aged asphalt, the free radical concentration in the system decreased significantly, and the oxidation induction period was prolonged obviously—further verifying the antioxidative efficacy of phenolic components. In terms of functional group-mediated depolymerization and structural regulation, aldehyde and ketone components, due to the strong electrophilicity of aldehyde groups, can selectively attack ether bonds and C-C single bonds in asphaltene macromolecular chains, triggering directional depolymerization reactions. This reduces the weight-average molecular weight (Mw) of asphaltenes and increases the aromatic content in the system from 18% to 25%. Deng et al. found through energy component analysis that bio-rejuvenators containing phenols, esters, acids, and amides outperform traditional petroleum-based rejuvenators (dominated by alkanes and aromatics) in the intercalation and depolymerization of oxidized asphaltenes. Specifically, the total system energy decreased significantly in the presence of typical phenolic components, and this energy change directly confirms the promotion effect of bio-rejuvenators on depolymerization reactions. DFT reactivity calculations by Mousavi et al. indicated that these chemicals have a low tendency to form new polar functional groups in the presence of oxidants; in contrast, asphaltene molecules exhibit the highest polarizability and are more prone to depolymerization [158]. Furthermore, carboxyl groups in bio-oil-based rejuvenators can form hydrogen bonds with hydroxyl groups of resins in aged asphalt, constructing a stable interfacial layer. This layer effectively inhibits the conversion of resins to asphaltenes, reducing the resin conversion rate by 40–50%. Differential Scanning Calorimetry (DSC) test results showed that for aged asphalt added with 10% palm oil fatty acid ester, the oxidative exothermic peak temperature of resins increased from 185 °C to 210 °C—indicating that the formation of the interfacial layer significantly improved the thermal stability of resins and reduced their deterioration at high temperatures. As shown in Figure 16, the infrared spectrum clearly displays the characteristic functional group absorption peaks of asphalt, including C-H stretching vibration, O-H stretching vibration, carbonyl and aromatic functional groups, etc. The differences in characteristic peaks of cis olefin, trans olefin and benzene ring related C-H bending vibration in SBS modified asphalt were compared through illustrations, providing a visual basis for the chemical structure characterization of asphalt and modified asphalt.

### 7.3. Compatibility and Dispersion Behavior

The compatibility between bio-based rejuvenators and aged asphalt is mainly achieved through solubility parameter matching and interfacial tension regulation, with the Hildebrand Solubility Parameter (HSP) theory as the core basis. The smaller the difference in solubility parameters between the rejuvenator and aged asphalt, the better the compatibility. For instance, the solubility parameter difference between lignin-based phenolic rejuvenators and aged asphalt is only 0.8–1.2 (J/cm^3^)^1/2^, significantly lower than that of petroleum-based rejuvenators (1.5–2.0 (J/cm^3^)^1/2^). Thus, they can disperse more uniformly in aged asphalt and avoid stratification. Jiao et al. investigated the compatibility at different temperatures and found that the optimal rheological performance is achieved at 160 °C.

From the perspective of interfacial tension, aldehyde and ketone components play a significant role. Without rejuvenators, the interfacial tension is approximately 25–30 mN/m; after adding 2–3% of the rejuvenator, it decreases to 15–20 mN/m. Centrifugation tests showed that the degree of stratification reduces from 12% to 4%, which is much lower than the 10% required by specifications. Meanwhile, molecular dynamics simulations indicated that cardanol has the smallest solubility parameter difference with aged asphalt and exhibits the most uniform distribution, while distilled tall oil enhances dispersion stability through the interaction of free fatty acids.

Gel Permeation Chromatography (GPC) tests also revealed that after adding bio-based rejuvenators, the content of large molecular size (LMS) components in aged asphalt decreases by 5.2%, and the molecular weight distribution becomes more uniform. Additionally, Jin et al. improved the compatibility by 126.76% through acid surface-activated crumb rubber. Cheng et al. also confirmed the influence of rejuvenators on the intermolecular interactions in the asphalt system through separation tests and fluorescence microscopy, providing microscopic references for compatibility regulation [160].

### 7.4. Mechanism of Regulating Asphalt Colloidal Structure

Bio-based rejuvenators achieve the transition from a “gel-type” to a “sol-type” structure by reconstructing the colloidal-network model of asphalt. This process is verified through SARA fraction regulation, micromorphological evolution, and mechanical property recovery [161,162].

From the perspective of SARA fractions, rejuvenators can adjust the proportions of saturates, aromatics, resins, and asphaltenes in aged asphalt: phenolic rejuvenators reduce asphaltene content by 8–12% and increase resin content by 5–8%; fatty acid ester-based rejuvenators increase aromatic content by 10–15% and saturate content by 3–5%. Ultimately, the Colloidal Stability Index is improved from <0.6 (unstable) to 0.8–1.0 (stable) [163].

In terms of micromorphology, Scanning Electron Microscopy observations show that the surface of aged asphalt has dense wrinkled structures. After adding bio-based rejuvenators, the wrinkle depth decreases from 200 to 300 nm to 50–100 nm, and the area ratio of “bee-like” structures drops from 35% to below 10%, indicating a more uniform asphalt colloidal structure [164]. Li et al. proposed that aged asphalt can recover its basic physical properties when the content of waste engine oil (WEO) is 1–5% and waste cooking oil (WCO) is 1–4% (by mass of asphalt). Further Atomic Force Microscopy (AFM) tests revealed that rejuvenators can repair the damaged “bee-like” structures in aged asphalt, reducing the root mean square roughness (Rq) from 2.910 nm to 1.315 nm and decreasing interfacial stress concentration areas. Macroscopically, this is reflected in the dynamic shear modulus (G*) decreasing from 11.4 kPa to 1.5 kPa, the phase angle (δ) increasing by 8.1°, and the viscoelasticity recovering to the level of virgin asphalt. Furthermore, dynamic mechanical analysis indicated that the crossover modulus of recycled asphalt increases from 5.86 × 10^6^ Pa to 1.16 × 10^7^ Pa, confirming the significant dispersion effect of asphaltene aggregates and the restored balance between elasticity and viscosity in the colloidal-network model. Ke et al. found that the solubility of saturates (long-chain and short-chain alkanes) increases with increasing concentration, thereby reducing the structural stability of the heavy oil colloidal system and inducing a gradual decrease in yield stress [165].

**Figure 17:**
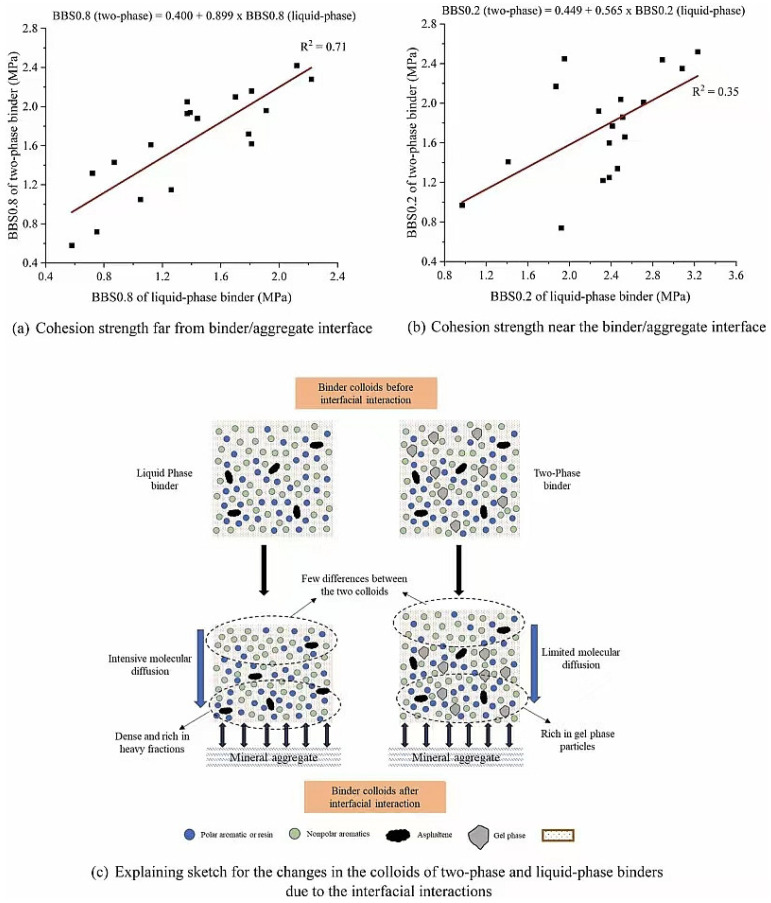
Response mechanism of colloidal interaction at the asphalt-aggregate interface [166].

Additionally, Mohamed et al. elucidated the regulatory mechanism of asphalt colloidal structure induced by asphalt-aggregate interfacial interactions, as illustrated in Figure 17. In the initial state, the colloidal composition difference between liquid-phase and two-phase binders is small; when interfacial interaction occurs with mineral aggregates, the liquid-phase binder undergoes intense molecular diffusion at the interface, forming a dense structure enriched in heavy fractions. In contrast, the molecular diffusion of the two-phase binder is restricted, and the interfacial region exhibits the characteristic of enrichment of gel-phase particles. This colloidal structure differentiation is directly reflected in the correlation of cohesive strength: far from the interface, the cohesive strengths of the two-phase and liquid-phase binders exhibit a strong linear correlation; close to the interface, the correlation weakens significantly. This clearly verifies the regulatory effect of interfacial interactions on asphalt colloidal structure and its associated impact on cohesive performance [166].

To summarize, bio-based rejuvenators improve fluidity through light fraction supplementation and segment intercalation, repair the aged chemical structure by involving active functional groups in free radical passivation and oxidation inhibition, and enhance colloidal dispersion and compatibility stability by relying on solubility parameter matching and interfacial energy regulation. These synergistic effects promote the transition of asphalt from a hard and brittle gel state back to a flexible sol state, significantly restoring its rheological properties and low-temperature ductility.

However, current research still has limitations, mainly reflected in three aspects: the contribution mechanisms of active components have not been fully quantified; the coupling laws between different aging stages and rejuvenator structures remain unclear; and systematic verification is lacking for the long-term evolution of rejuvenation effects and resistance mechanisms against re-aging. Moving forward, it is necessary to strengthen the integration of multi-scale coupled characterization and molecular simulation and establish a molecular-oriented design system based on functional group design and solubility parameter regulation, thereby providing a scientific basis for the long-term application of bio-based rejuvenators in high-content recycled asphalt systems.

## 8. Challenges Facing Bio-Based Asphalt Rejuvenators

### 8.1. Analysis of Existing Scientific Bottlenecks

The core scientific bottlenecks restricting the application of bio-based rejuvenators in the asphalt field can be summarized into four core dimensions: (a) compatibility issues of rejuvenators, (b) complexity of asphalt aging mechanisms, (c) inconsistent effects of different rejuvenators on asphalt performance, and (d) unclear synergistic mechanism between rejuvenators and recycled aggregates, warm mix asphalt (WMA) technology, and modifiers.

In terms of rejuvenator compatibility, no quantitative regulation mechanism has been established for the molecular-level compatibility between bio-oil and the colloidal system of aged asphalt, as well as the interfacial adhesion stability with aggregates. In particular, the interfacial failure mechanism under extreme conditions such as humid environments and heavy traffic loads remains unclear. The complexity of the aging mechanism is reflected in the fact that field asphalt aging is a dynamic evolution process under the multi-field coupling of heat, oxygen, ultraviolet radiation, and traffic loading. However, most existing studies rely on laboratory single-factor aging simulation, which is difficult to truly restore the evolution law of molecular structure during field aging [167,168]. The inconsistent influence of different rejuvenators on asphalt performance originates from the heterogeneity of the chemical composition of biomass feedstocks. Differences in the type and content of functional groups such as fatty acid methyl esters (FAME) and phenolic hydroxyl groups directly lead to significant differentiation in the key performance of rejuvenated asphalt, including low-temperature cracking resistance, high-temperature rutting resistance, and anti-aging performance [169]. In addition, there is a lack of systematic quantitative research on the synergistic mechanism between rejuvenators and recycled aggregates, WMA technology, and modifiers. The residual aged asphalt film on the surface of recycled aggregates will significantly affect the permeation and diffusion of rejuvenators; the low-temperature environment in WMA production may inhibit the activity of rejuvenators; and the uncontrolled chemical reactions between bio-based rejuvenators and modifiers such as styrene-butadiene-styrene (SBS) may damage asphalt performance. The critical conditions and regulation laws of these synergistic effects are still unclear, which further aggravates the technical bottlenecks [170].

### 8.2. Analysis of Existing Research Defects and Controversies

The defects and contradictions in existing studies are fourfold. First, the one-sided understanding of the rejuvenation mechanism. Most studies attribute the effect of bio-based rejuvenators to physical miscibility and light fraction supplementation, ignoring the chemical regulation potential such as hydrogen bonding between polar functional groups and asphaltenes, and free radical scavenging, resulting in a deviation between the theoretical prediction and actual performance of the rejuvenation effect [171]. Second, the limitations of performance evaluation. Most laboratory studies focus on short-term rheological properties, while ignoring the attenuation law of pavement performance including long-term aging, moisture damage, and fatigue cracking. This makes it difficult to translate the recovery effect observed in the laboratory into field durability, and there is a particular lack of accurate prediction of the performance evolution of rejuvenated asphalt under multi-field coupled aging. Third, the misleading nature of overgeneralized conclusions. Some studies extrapolate the rejuvenation effect of a specific bio-oil to all bio-based rejuvenators, ignoring the decisive influence of feedstock sources and pretreatment processes on rejuvenation performance, leading to contradictory conclusions among different studies. Fourth, the lack of in-depth research on microscopic mechanisms. Most existing studies infer the mechanism from macroscopic performance, lacking long-term dynamic monitoring at the molecular and microscopic scales. It is difficult to establish the correlation between aging factors, structural changes, and performance attenuation, and there are still controversies over the core mechanism of rejuvenation durability, which hinders the proposal of targeted improvement strategies [172].

### 8.3. Critical Review and Analysis of Existing Research Literature

Criticisms of existing literature mainly focus on the simplicity of research methods, limitations of the evaluation system, unreliability of conclusions, and lack of research on synergistic mechanisms. In terms of research methods, most studies use standardized laboratory aging tests to simulate field aging, without considering the differences in aging kinetics under multi-field coupling. In addition, parameters such as rejuvenator dosage and mixing process are mostly set based on empirical experience, lacking systematic optimization. In terms of the evaluation system, the existing index system adopts the evaluation framework developed for petroleum-based rejuvenators, and no special evaluation method has been established for the environmental characteristics and long-term performance attenuation of bio-based rejuvenators, resulting in incomplete performance evaluation. In particular, the influence of the synergistic effect between rejuvenators, recycled aggregates, WMA technology, and modifiers on pavement performance is ignored. In terms of conclusion reliability, some studies do not fully consider the variability of biomass feedstocks, over-extrapolate conclusions from small-sample tests, and lack support from long-term engineering tracking data, making it difficult to verify the stability and durability of the rejuvenation effect. In terms of synergistic mechanism research, most existing studies focus on the effect of a single rejuvenator, and no compatibility design criteria for the ternary system of rejuvenator, recycled aggregate, and WMA technology/modifiers have been established, making it difficult to achieve the synergistic improvement of the high- and low-temperature performance of rejuvenated asphalt.

### 8.4. Tracing Analysis of Divergences in Research Results

The divergences in research results essentially originate from the heterogeneity of bio-based rejuvenators, the complexity of application scenarios, and the uncertainty of synergistic effects. Different types of bio-oil exhibit different effects in restoring the basic properties of aged asphalt and may also show differences in specific application environments. Existing rejuvenators have significant differences in effectiveness in restoring the low-temperature performance of aged SBS-modified asphalt, especially for severely aged binders. Different types of bio-oil show obvious performance differentiation in rejuvenated asphalt: some bio-oils have excellent performance in improving low-temperature cracking resistance but have limited effect on high-temperature rutting resistance. Studies have shown that the composition and properties of bio-oil have a critical impact on its rejuvenation effect. Differences in parameters such as fatty acid composition, polar functional group content, and molecular weight distribution directly lead to the performance differentiation of rejuvenated asphalt. Meanwhile, differences in application conditions, including the aging degree of the asphalt binder, aggregate gradation, and construction technology, will also amplify the difference in rejuvenation effects. In addition, the synergistic effect between rejuvenators, recycled aggregates, WMA technology, and modifiers is affected by multiple factors, such as the thickness of the residual asphalt film on recycled aggregates, the temperature reduction amplitude in WMA production, and the type of modifiers. The combined differences in these factors make it difficult to unify the conclusions of different studies, further aggravating the divergence of research results.

### 8.5. Restricting Factors for Large-Scale Engineering Application

The large-scale application of bio-based rejuvenators is restricted by four core aspects: quality stability, large-scale preparation, performance durability, and synergistic mechanism.

In terms of quality stability, the inherent heterogeneity of biomass feedstocks is the fundamental inducing factor. Whether it is vegetable oils, algae, or agricultural and forestry wastes, their composition fluctuates significantly with variety, growth environment, and harvesting time. For example, the free fatty acid content of palm oil from different producing regions can vary by up to 5–8%, and the lignin content of corn stover can also fluctuate in the range of 6–10%. Such feedstock differences directly lead to the difficulty in unifying the content of active components such as fatty acid esters and phenols in rejuvenators, which further causes performance deviation of different batches of rejuvenators.

In the large-scale preparation process, process adaptability and cost control are two major difficulties. Processes commonly used in the laboratory, such as microwave-assisted pyrolysis and enzymatic transesterification, are mostly based on small-batch precise control mode, and problems will occur once scaled up. For example, microwave-assisted pyrolysis can achieve a high bio-oil yield in the laboratory, but the yield is significantly reduced in large-scale production due to the non-uniformity of the microwave field. Although enzymatic transesterification can reduce saponification side reactions, the high cost of enzyme preparations and their low reusability lead to an increase in the production cost of rejuvenators [173]. In addition, the collection and pretreatment of biomass feedstocks are also challenging. The collection cost of scattered agricultural and forestry residues such as straw and branches accounts for a high proportion of the total raw material cost. At present, only feedstocks with relatively stable sources such as waste cooking oil have achieved small-batch production, and other types of bio-based rejuvenators are still in the laboratory stage [174].

The problem of performance durability has not been fundamentally solved. The mechanism of long-term aging and rejuvenation durability of rejuvenated asphalt has not been clearly elucidated. Most existing studies focus on short-term single-factor aging tests, lacking accurate prediction of the performance attenuation law under multi-field coupled aging. There are still controversies over the core mechanism of rejuvenation durability, and the lack of long-term engineering tracking data makes it difficult to verify the stability and durability of the rejuvenation effect, which cannot meet the requirements of engineering applications for long-term service performance.

For the synergistic mechanism bottleneck, the synergistic mechanism between rejuvenators, recycled aggregates, WMA technology, and modifiers is still unclear. The residual asphalt film on recycled aggregates will affect the permeation and diffusion of rejuvenators; the low-temperature environment in WMA production may inhibit the activity of rejuvenators; and the uncontrolled chemical reactions between bio-based rejuvenators and SBS modifiers may damage asphalt performance. At present, there is a lack of targeted adaptability optimization schemes and compatibility design criteria for these synergistic effects, making it difficult to maximize the comprehensive performance of rejuvenated asphalt, which further restricts its engineering application [175].

## 9. Conclusions

This review systematically elaborates on the core values of bio-based rejuvenators—rooted in multi-source raw material systems and multi-path synergistic regeneration mechanisms—in repairing the colloidal structure of aged asphalt, optimizing full-temperature-range performance, and advancing the low-carbon paradigm. Consequently, it provides a scientific foundation and technical pathway for replacing petroleum-based rejuvenators.

1. Biomass materials such as vegetable oils, lignin derivatives, and algal oils can be converted into rejuvenators via processes like transesterification and pyrolysis. Optimizing these processes enables the regulation of active components and the enhancement of stability. Currently, there exist problems including raw material heterogeneity, poor adaptability of large-scale processes, and high energy consumption in pretreatment. Future research should focus on raw material standardization, continuous production, and the development of low-energy-consumption pretreatment technologies.
2. The core feature of bio-based rejuvenators lies in their amphiphilic structure, containing multiple types of active components such as fatty acid esters. These rejuvenators can interact specifically with the SARA fractions of asphalt to repair the colloidal structure. At present, the quantitative evaluation of the contribution of active groups is insufficient, and the impact of impurities remains unclear. Future studies need to establish quantitative characterization models and combine multi-scale simulations to deepen the research on structure-performance relationships.
3. Asphalt aging is an irreversible process involving the loss of light fractions and the aggregation of asphaltenes, leading to performance degradation. Bio-based rejuvenators can restore asphalt performance by supplementing lost fractions and reconstructing the colloidal structure. Existing studies have insufficient simulation of coupled aging and unclear molecular mechanisms. Future research should construct a multi-environmental aging simulation system and establish a correlation model between microscale and macroscale performance.
4. Bio-based rejuvenators can improve the rheological properties of asphalt, optimize high-low temperature performance and fatigue resistance. Rejuvenators derived from different raw materials exhibit distinct performance advantages and superior environmental friendliness. However, there are challenges such as the trade-off between high and low temperature performance and insufficient verification of long-term durability. In the future, performance optimization should be achieved through compounding, and long-term service monitoring in engineering scenarios should be strengthened.
5. The regeneration effect of bio-based rejuvenators is achieved through the synergistic action of multiple pathways, including physical dilution, chemical complementarity, compatibility regulation, and colloidal reconstruction. Molecular simulations have confirmed their microscopic mechanisms. Currently, the understanding of aging-regeneration coupling laws is unclear, and the long-term evolution mechanism remains ambiguous. Future research needs to integrate multi-scale characterization and simulation technologies to reveal the action mechanisms under complex environments.
6. Bio-based rejuvenators are confronted with multiple challenges, such as quality stability, large-scale production, and insufficient synergistic effects with other materials/technologies. In the future, efforts should be made to develop customized products through intelligent design, improve the standardization system, strengthen the synergistic adaptability with warm mix asphalt technology and recycled aggregates, promote their transformation from laboratory research to large-scale engineering applications, and support the development of low-carbon transportation.

## Figures and Tables

**Table 2:** Process parameters for preparing lignin-based bio-oil.

Researcher	Production Technology	Processing Parameters
Kanaujia et al. [44]	Thermochemical pyrolysis of lignocellulosic biomass—bio-oil refining	Pyrolysis temperature > 500 °C, in an oxygen-free environment; bio-oil yield is 70–80%
Dai et al. [45]	Biomass pretreatment—catalytic pyrolysis	The pretreatment temperature ranges from 200 to 300 °C, while the catalytic temperature spans from 450 to 600 °C. The catalysts used are HZSM-5 and Ga/ZSM-5
Dai et al. [46]	Baking—co-pyrolysis	Baking temperature: 220–280 °C; Co-pyrolysis temperature: 500–550 °C; Ratio of biomass to HDPE: 1:1
Kumar et al. [47]	Acid pretreatment—microwave-assisted pyrolysis	Acid concentration: 0.1–5 wt% (H_2_SO_4_), microwave power: 300–600 W, pyrolysis temperature: 450–550 °C

**Table 3:** Summary of performance improvements.

Researcher	Oil	Protocol	Performance Index Improvement
Li et al. [130]	Epoxidized soybean oil	DSR, BBR, PG, Aging	Low temperature performance, high temperature performance, and PG enhancement, with significantly improved fatigue resistance
Li et al. [131]	Epoxidized soybean oil	DSR, BBR, AFM, PG	Low temperature performance, high temperature performance, and PG enhancement have significantly improved fatigue resistance
Zhang et al. [132]	Epoxidized soybean oil	DSR, BBR, PG, Aging	The fatigue resistance is significantly improved; the low-temperature performance is positively correlated with the oil content and negatively correlated with the epoxy number
Felipe et al. [133]	Soybean oil	DSR, BBR, PG	Low temperature performance, high temperature performance, and PG improvement, with significant enhancement in fatigue resistance
Zhang et al. [134]	Soybean oil	DSR, BBR, Molecular dynamics simulation	The stability has been significantly improved
Gao et al. [135]	Palm oil	DSR, BBR	The high-temperature performance is slightly lower than that of RAP but higher than expected, while both the low-temperature performance and fatigue performance have been improved
Antonia et al. [136]	Palm oil	DSR, BBR, TGA	The low-temperature performance has been improved, and the specific impact depends on the type and temperature
Zhang et al. [137]	Straw oil	DSR, BBR, TGA	High-temperature performance and fatigue resistance are improved, but low-temperature performance is sacrificed
Zhang et al. [138]	Straw oil	DSR, BBR	The low-temperature performance is significantly improved, and the softening point decreases as the dosage increases
Zhang et al. [139]	Straw oil	DSR, BBR	The low-temperature performance and fatigue resistance have been improved, while the high-temperature performance has been restored
Yue et al. [140]	Straw oil	DSR, BBR, AFM	The low-temperature performance and fatigue resistance have been significantly improved, and the microstructure has been enhanced
Ma et al. [141]	Straw oil	DSR, BBR	High temperature performance and fatigue resistance are significantly improved
Wang et al. [142]	Castor oil, Straw oil, Gutter oil	DSR, BBR	The high-temperature performance is significantly improved when using castor oil and straw oil. The low-temperature performance is significantly improved when using gutter oil
Alireza et al. [143]	Algae oil	DSR, BBR, Molecular dynamics simulation	Low-temperature performance and high-temperature performance have been restored
Li et al. [144]	Bio-heavy oil	DSR, BBR	At a specific content, the low-temperature performance is significantly improved. However, when it exceeds a certain content, the high-temperature performance is sacrificed
Lv et al. [145]	Vegetable oil	DSR, BBR, SEM	Low-temperature performance and high-temperature performance are improved, and anti-aging ability is significantly enhanced
Gong et al. [146]	Biodiesel produced from WCO as raw material	DSR, BBR, RV	At a specific content, the low-temperature performance is significantly improved, while the high-temperature performance is sacrificed

## Data Availability

No new data were created or analyzed in this study. Data sharing is not applicable to this article.
